# Omics Approaches to Study Formation and Function of Human Placental Syncytiotrophoblast

**DOI:** 10.3389/fcell.2021.674162

**Published:** 2021-06-15

**Authors:** Adam Jaremek, Mariyan J. Jeyarajah, Gargi Jaju Bhattad, Stephen J. Renaud

**Affiliations:** ^1^Department of Anatomy and Cell Biology, Schulich School of Medicine and Dentistry, University of Western Ontario, London, ON, Canada; ^2^Children’s Health Research Institute, Lawson Health Research Institute, London, ON, Canada

**Keywords:** pregnancy, placenta, trophoblast, syncytiotrophoblast, omics, cell models

## Abstract

Proper development of the placenta is vital for pregnancy success. The placenta regulates exchange of nutrients and gases between maternal and fetal blood and produces hormones essential to maintain pregnancy. The placental cell lineage primarily responsible for performing these functions is a multinucleated entity called syncytiotrophoblast. Syncytiotrophoblast is continuously replenished throughout pregnancy by fusion of underlying progenitor cells called cytotrophoblasts. Dysregulated syncytiotrophoblast formation disrupts the integrity of the placental exchange surface, which can be detrimental to maternal and fetal health. Moreover, various factors produced by syncytiotrophoblast enter into maternal circulation, where they profoundly impact maternal physiology and are promising diagnostic indicators of pregnancy health. Despite the multifunctional importance of syncytiotrophoblast for pregnancy success, there is still much to learn about how its formation is regulated in normal and diseased states. ‘Omics’ approaches are gaining traction in many fields to provide a more holistic perspective of cell, tissue, and organ function. Herein, we review human syncytiotrophoblast development and current model systems used for its study, discuss how ‘omics’ strategies have been used to provide multidimensional insights into its formation and function, and highlight limitations of current platforms as well as consider future avenues for exploration.

## Introduction

The placenta is a temporary organ that forms during pregnancy. It serves crucial functions to sustain pregnancy, promote fetal growth and development, and stimulate adaptive changes in maternal physiology and metabolism. These functions include (but are not limited to) hormone production and metabolism, hemodynamic adaptations, and serving as a physical barrier separating maternal and fetal circulations. The placental barrier enables nutrients, gases, and wastes to diffuse between maternal and fetal blood, yet protects the fetus from potentially harmful factors including toxins, pathogens, and maternal immune reactivity. Structural adaptations have evolved to enable the placental barrier to execute its versatile requirements as both protector and nurturer, culminating in the formation of a unique multinucleated syncytium consisting of millions of nuclei connected by a continuous cytoplasm, called syncytiotrophoblast (STB). In humans, STB facilitates implantation and ultimately lines chorionic villi where it bathes in maternal blood. STB secretions and debris are deposited into the maternal circulation, where they have important roles in modulating maternal physiology as well as diagnostic potential for fetal-placental aberrations and pregnancy disease. Despite the importance of STB formation and function for fetal development and pregnancy outcome, limitations of cell and animal models have left much to be discovered. In the first part of this review, we will briefly highlight the ontogeny and diverse functions of human STB as well as models commonly used for its study. Then, we will discuss how various omics technologies have provided unprecedented insights into understanding STB formation and function, including current limitations, challenges, and opportunities for future investigation.

## Ontogeny of STB

**FIGURE 1:**
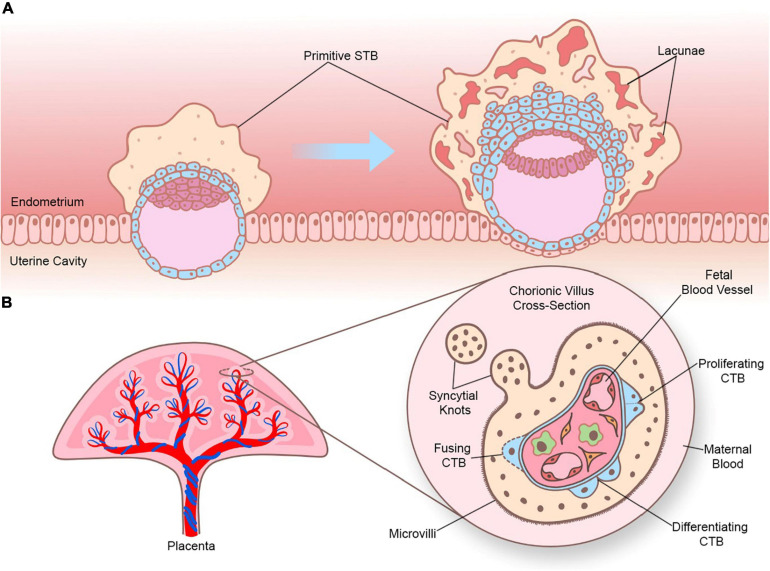
Sequential development of STB. **(A)** Progression of a human embryo at approximately gestational days 7–8 (peri-implantation; left image) and days 9–10 (post-implantation; right image). Please note the invasive properties of the primitive STB at the forefront of the implanting human embryo and the gradual development of blood-filled lacunae. **(B)** Cross section of a chorionic villus in later gestation. Please note that the STB layer exhibits apical-basal polarity (as shown by the presence of microvilli) and bathes directly in maternal blood. An extruding syncytial knot is also shown. The villus core contains blood vessels that connect to the fetal circulation, as well as different cell types (such as Hofbauer cells and fibroblasts). CTBs are shown in various stages of their life cycle (proliferating, differentiating, and fusing).

There are two types of STB that arise during different stages of human embryogenesis: a primitive STB that mediates implantation and decidual erosion during the second week after fertilization, and a definitive STB that lines chorionic villi from the third week and beyond. Whether these two STB subtypes are distinct entities or the gradual evolution of the same lineage as gestation progresses is unclear. The primitive STB first appears around the time of implantation as the blastocyst breaches the uterine surface epithelium, likely through intercellular fusion of underlying cytotrophoblasts (CTBs) at the embryonic pole of the blastocyst. The primitive STB rapidly expands into the decidua and erodes uterine stroma, glands, and capillaries. The cavities generated within the primitive STB, called lacunae, become filled with blood and glandular secretions from eroded decidual tissue, providing a source of early nutrition for the conceptus. The primitive STB provides the groundwork in which pillars of CTBs proliferate, forming primary villi. These primary villi traverse the entirety of the primitive STB and ultimately connect together to encircle the conceptus as the CTB shell, which serves to anchor the conceptus to the decidua basalis. Villi also branch extensively to create smaller floating villi that remain within spaces between villi (intervillous spaces), increasing the surface area of the villus tree. Chorionic villi are formed when extraembryonic mesoderm and blood vessels emanating from the allantois infiltrate the proximal cores of the primary villi during the third week of development. Thus, the villus core includes an inner meshwork of mesoderm-derived stroma consisting of fibroblasts and immune cells (notably macrophages) as well as blood vessels that are contiguous with the fetal circulation via the umbilical vessels. The core is lined by a trophoblast bilayer containing an outer STB layer and an inner CTB layer, which are physically separated from the stroma by a laminin-rich basement membrane. Blood vessels known as spiral arteries course through the decidua basalis and supply maternal blood to the intervillous spaces. Since STB lines the outer surface of the villi, it directly bathes in maternal blood and forms a key site of interaction between maternal and fetal tissue. A schematic illustrating the primitive and definitive STB is presented in Figure 1.

## Differentiation of CTB Into STB

Syncytiotrophoblast has a limited lifespan and must be regularly replenished throughout pregnancy with fresh cytoplasm and nuclei by controlled differentiation and fusion of underlying CTBs. Differentiation and fusion of CTBs into STB is a complex and highly orchestrated process that involves biochemical changes to support the immense endocrinological and secretory functions of STB as well as morphological changes to enable intercellular fusion. It is not yet clear whether the signal that initiates these biochemical and morphological events originates from the STB or from the underlying CTB layer. Additionally, while biochemical and morphological differentiation are coupled, they are thought to be executed by discrete pathways (Orendi et al., 2010). Biochemical differentiation requires that CTBs exit the cell cycle (Lu et al., 2017), and repress genes involved in maintaining a progenitor state, such as *ELF5*, *TP63*, *ID2*, and *TEAD4*. Simultaneously, factors implicated in nutrient transport, immunomodulation, and hormone biosynthesis and metabolism are induced. The integration of multiple signaling pathways and transcription factors (TFs) are implicated in the process of CTB differentiation, including suppression of WNT and activin/transforming growth factor beta (TGF-β) signaling as well as activation of cyclic adenosine monophosphate (cAMP)/protein kinase A (PKA) and mitogen-activated protein kinase (MAPK) pathways. CTB differentiation also involves the activity of many TFs and epigenetic regulators including PPARG, DLX3, GCM1, TFAP2A, OVOL1, and many others. A detailed characterization of TFs implicated in trophoblast differentiation is discussed in several comprehensive reviews (Knott and Paul, 2014; Baines and Renaud, 2017; Knöfler et al., 2019).

Morphologically, CTB fusion necessitates a modified epithelial-mesenchymal transition resulting in loss of junctional proteins such as E-cadherin, reorganization of cytoskeletal components, and intercellular mixing of intracellular contents (Ishikawa et al., 2014). The impetus for intercellular fusion is largely mediated by expression of cellular fusogens called syncytins. Syncytins are encoded by co-opted endogenous retroviral (ERV) envelope genes *ERVW-1* (encodes syncytin-1) and *ERVFRD-1* (encodes syncytin-2). Syncytin-1, which is expressed in STB, binds to the neutral amino acid transporter ASCT-2 expressed mainly by CTBs. Syncytin-2, on the other hand, is expressed in small clusters of CTBs and binds to MFSD2A, which is expressed by STB (Lavialle et al., 2013). Additionally, changes in the cytoskeleton are required to form the extensive microvilli that cover the apical surface of STB and increase the surface area of the STB up to sevenfold (Teasdale and Jean-Jacques, 1985).

## STB Life Cycle

Syncytiotrophoblast undergoes highly regulated turnover as aged or damaged syncytia are replaced by newly formed ones through fusion of underlying CTBs (Gauster et al., 2009). Since this occurs continuously from implantation until term, the nuclei present in STB are of different ages and exhibit a range of morphologies and packing densities that reflect progressive maturation. Within STB, clustering of nuclei occurs in regions known as syncytial sprouts and knots (Mayhew, 2014). Syncytial sprouts, which are predominant during the first-trimester, harbor nuclei that are primarily euchromatic with a distinct nucleolus. They form protrusions in the development of new villi, yet their connection with the villus surface can become attenuated and render them susceptible to detachment and release into the intervillous space (Burton, 2011). Syncytial knots, which often protrude from the surface of villi during the third trimester, contain more densely clustered nuclei that may be less transcriptionally active based on features such as dense condensations of heterochromatin and lack of apparent nucleoli (Burton and Jones, 2009). Although the nuclei resemble those classified as apoptotic, whether syncytial knots represent an apoptotic end-stage of the STB life cycle remains elusive as nuclear fragmentation is not observed (Mayhew, 2014). Nevertheless, knots are considered a means by which aged STB nuclei are sequestered to regions of the villus membrane where they do not interfere with exchange (Fogarty et al., 2013), and some normally detach to be shed into maternal circulation (Mayhew et al., 1999). The volume of syncytial knots relative to CTB volume increases during gestation, suggesting that early proliferation is geared toward growth with later proliferation toward renewal and repair (Mayhew and Barker, 2001).

Over the course of pregnancy, STB releases a variety of factors into maternal circulation that are critical for the maintenance of healthy pregnancy. This includes fragments derived from syncytial sprouts or knots, which range from small subcellular particles to large multinucleated fragments, that may play important roles in maintaining maternal immune tolerance to fetal tissues (Chamley et al., 2011). Furthermore, STB releases membrane-bound vesicles known as STB extracellular vesicles (STBEV) in the form of exosomes, microvesicles, or apoptotic bodies, from the villus surface into maternal circulation (Tannetta et al., 2017a). These vesicles contain a variety of biologically active molecules, such as proteins, RNAs, and lipids, that have regulatory roles in the maternal immune response to pregnancy and may interact with components of maternal circulation, such as endothelial cells or leukocytes, to facilitate maternal-fetal communication (Tannetta et al., 2017b). STB also releases cell-free ‘fetal’ DNA (cfDNA) into maternal blood that varies in concentration based on multiple factors including oxidative stress (Taglauer et al., 2014). Additional factors that are produced and released by STB include numerous steroid and peptide hormones, such as estrogen, progesterone, human chorionic gonadotropin (hCG), human placental lactogen (hPL), and placental growth hormone (PGH) (Murphy et al., 2006). STB also produces a variety of growth factors, such as pregnancy-specific glycoproteins (PSGs), vascular endothelial growth factor (VEGF), placental growth factor (PlGF), TGF-β, and many other cytokines, chemokines, and signaling molecules (Kidima, 2015).

## STB Formation in Pregnancy Disease

Abnormal formation or function of STB during pregnancy is implicated in the etiology of pregnancy complications, such as preeclampsia and fetal growth restriction (FGR). Preeclampsia is a serious disease characterized by vascular damage and hypertension in the mother during the latter half of pregnancy that can result in further organ deficiency and damage. Currently, the only definitive treatment is to remove the placenta and therefore deliver the baby, which can lead to complications associated with prematurity if performed prior to 37 weeks. FGR is the failure of a fetus to achieve its growth potential as predetermined by genetic and epigenetic factors (Burton and Jauniaux, 2018). Low birth weight as a result of FGR or premature delivery increases risk of perinatal death and morbidity and predisposes the child to lifelong risk of developing serious chronic diseases. Cultured CTBs from preeclampsia or FGR-affected placentas show impaired cell fusion and reduced expression of key fusion mediators (Langbein et al., 2008). In STB from these placentas, there is a greater number of apoptotic nuclei present (Ishihara et al., 2002). Preeclampsia is also associated with increased syncytial knotting as well as greater extrusion of STB fragments and pro-inflammatory STBEVs that are implicated in immune dysregulation and endothelial damage (Roland et al., 2016; Tannetta et al., 2017b). In addition, there is altered composition of placental proteins within STBEVs isolated from plasma of women with pregnancy-related disorders such as preeclampsia, which holds promise to be exploited as potential biomarkers for early diagnosis and monitoring (Levine et al., 2020). Current screening methods that use maternal serum biomarkers of STB stress, such as increased soluble vascular endothelial growth factor receptor 1 (sFLT1) and endoglin or reduced PlGF, are limited as these changes may not appear in later onset forms of disease with no early STB pathology (Redman and Staff, 2015).

## Models to Study STB Development

Models commonly used to study human STB are listed in Table 1. Although this review will focus on human STB, it is noteworthy that animal models with a syncytialized placental barrier (including rodents and primates) have been instrumental in providing insight into STB formation and function. In many cases, factors identified as critical for STB formation in animal models have subsequently been shown to have a conserved function in human STB development.

**TABLE 1:** Cell models used to study STB development and function.

Source	Cell model	Reference(s)	Notes
Placenta	Placental villus explants	Miller et al., 2005; Baczyk et al., 2006	Prepared by dissecting placental tissue and incubating in tissue culture wells for defined time periods. Denudation and regeneration of STB is also possible. Benefits include preservation of tissue integrity. Challenges include minimizing variation within and between experiments due to heterogeneity of explant preparation.
	Placental (CTB) organoids	Haider et al., 2018; Turco et al., 2018	Derived from early gestation placentas and can be expanded and cultured long-term. Provides a powerful model to study STB formation in 3D. Of note, the STB layer faces toward the inside of the organoid, so modeling transplacental passage of substrates may be limited.
	hTSCs	Okae et al., 2018	Derived from early gestation placentas or human blastocysts. Can be maintained as CTB-like cells in the presence of GSK-3, TGF-β, and HDAC inhibitors. Cells form STB-like cells after removing these inhibitors and adding forskolin.
	Primary CTBs	Kliman et al., 1986; Petroff et al., 2006	Isolated and enriched from placentas following delivery. Advantageous because they have undergone few population doublings or manipulations, and spontaneously form STB in culture. Cells have limited capacity to proliferate in culture, so they are less well suited to study early stages of syncytialization. Contamination with unwanted cell types and changes in CTB viability after isolation can pose a challenge.
Choriocarcinoma	BeWo	Pattillo and Gey, 1968	Cells have extended lifespans in culture. Beneficial for studying molecular aspects of cell fusion and hormone production, but possess genetic signatures distinct from normal trophoblast, so results should be interpreted with caution. Exposing BeWo cells to cAMP agonists stimulates STB-like cell fusion and hormone production. JEG-3, JAR, and ACH3P produce hormones (hCG) in response to cAMP agonists, but do not fuse, so their utility for modeling STB formation is limited. JEG-3 cells form STB-like cells when placed in 3D culture with microvascular cells.
	JEG-3	Kohler and Bridson, 1971	
	JAR	Pattillo et al., 1971	
	ACH3P	Hiden et al., 2007	
Early-stage embryos	hESCs	Xu et al., 2002; Amita et al., 2013	Using defined culture conditions, hESCs and hPSCs differentiate into cells possessing trophoblast-like properties, including STB-like cells. Beneficial for studying normal and pathological STB development from distinct genetic backgrounds, although there is contention about whether these cells truly represent trophoblast. Cells derived from naïve hESCs and hPSCs (rather than primed hESCs and hPSCs) appear to form bona fide trophoblast and can delineate the entire trophoblast developmental trajectory from pre- to post-implantation, including STB formation.
Reprogrammed somatic cells	Primed hPSCs	Chen et al., 2013; Horii et al., 2016; Wei et al., 2017	
	Naïve hPSCs	Dong et al., 2020; Guo et al., 2021; Io et al., 2021	

Since the placenta is expelled at the end of pregnancy (i.e., early pregnancy termination or delivery) and is often considered clinical waste, it is possible to conduct experiments using placental tissue. Moreover, unlike many other tissues used for *ex vivo* analyses that are biopsied or removed only when diseased, it is possible to collect placental tissue from pregnancies deemed healthy. To study STB biology, villus explants can be cultured for defined time periods, which is advantageous to study STB function while preserving tissue integrity (Miller et al., 2005). CTBs can also be isolated and enriched from placental tissue (Kliman et al., 1986). Isolated CTBs spontaneously differentiate into STB following removal from intact tissue and are considered a reliable representation of STB generation. Since isolated CTBs have limited capacity for proliferation in culture, they are not well suited for mechanistically studying repression of proliferation during early stages of STB formation.

Choriocarcinoma cell-lines are a valuable tool to study STB biology due to their resiliency and extended lifespan in culture. BeWo cells were derived from a brain metastasis, serially cultivated, and are adapted to cell culture (Pattillo and Gey, 1968). Differentiation of BeWo cells into hormone-producing STB-like cells is stimulated following exposure to agents such as forskolin (Wice et al., 1990). Forskolin activates adenylate cyclase, which increases intracellular levels of cAMP, thereby stimulating cAMP-sensitive pathways implicated in STB generation such as PKA (Gerbaud et al., 2015). Other commonly used choriocarcinoma cells, including JEG-3, JAR, and ACH3P, produce hCG in response to forskolin, but do not fuse under standard culture conditions (Borges et al., 2003; Rothbauer et al., 2017). Thus, their utility as models of STB development is limited.

In 2018, culture conditions to maintain trophoblast stem cells (TSCs) from human embryos or first-trimester placentas were determined (Okae et al., 2018). These cells can be maintained as CTB-like cells or stimulated to form STB-like cells. Organoid cultures of human trophoblasts derived from first-trimester placenta have also been established, which provide a powerful model to study human STB biology while considering three-dimensional (3D) spatial configuration (Haider et al., 2018; Turco et al., 2018).

Human embryonic stem cells (hESCs) and pluripotent stem cells (hPSCs) cultured under defined culture conditions differentiate into cells with features consistent with trophoblasts, including STB (Xu et al., 2002; Amita et al., 2013; Li et al., 2019). In particular, recent reports indicate that naïve hPSCs can be used to model the entire trophoblast lineage trajectory from trophectoderm through CTBs to STB (Dong et al., 2020; Guo et al., 2021; Io et al., 2021). STB derived from hESCs and hPSCs offer the possibility of studying normal and pathological STB development from distinct genetic backgrounds. For instance, defective STB formation in placentas with trisomy-21 can be recapitulated using trisomy-21 hPSCs (Horii et al., 2016).

## Omics: an Overview

**FIGURE 2:**
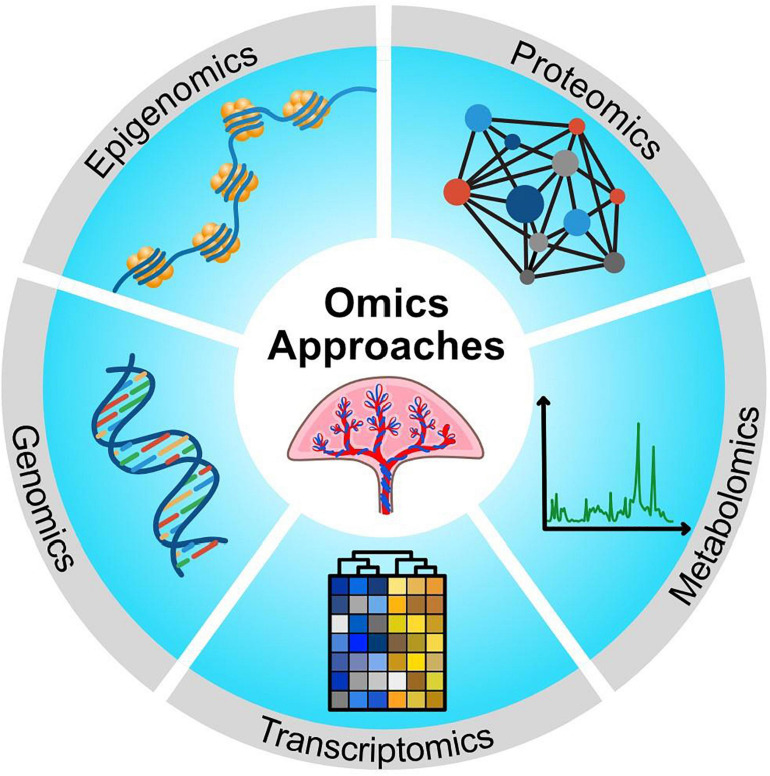
Schematic showing multiple omics approaches that have been used to make new discoveries about STB biology.

‘Omics’ technologies provide a holistic and integrative approach toward the study of biological systems. To obtain a systems level understanding of biology and disease, large-scale data on DNA, RNA, protein, and/or metabolites are produced, which are then organized by computational tools to provide a framework for the hierarchical contribution of integrated cellular pathways (Debnath et al., 2010; Karahalil, 2016). The term ‘omic’ derives from the suffix –ome, which means ‘whole’, and is added to terms like gene, transcript, and protein to create names that encompass the entire set of biological molecules in a system, such as genome, transcriptome, and proteome. The addition of ‘omics’ to the terms (genomics, transcriptomics, and proteomics) refers to the comprehensive assessment of these molecules in a non-targeted and unbiased manner (Nalbantoglu and Karadag, 2019). An overview of omics approaches used to study STB biology is provided in Figure 2.

Genomics is the study of the genome and the genetic basis underlying disease. The emergence of high-throughput methods, such as genotype arrays and next-generation sequencing (NGS), have enabled large-scale analyses of DNA sequences to identify copy number variations, small insertions and deletions, as well as single nucleotide variations between individuals, tissues, or cells (Hasin et al., 2017). Epigenomics focuses on the genome-wide characterization of reversible, and sometimes heritable, modifications of DNA or DNA-associated proteins resulting in altered chromosome conformation or gene expression without changes in the DNA sequence. Epigenomics approaches include global assessment of DNA methylation through NGS following bisulfite treatment of DNA, profiling chromatin accessibility (e.g., assay for transposase-accessible chromatin coupled to sequencing, ATAC-seq), chromosome conformation capture technologies, or the characterization of DNA-binding protein distribution through chromatin immunoprecipitation followed by sequencing (ChIP-seq) (Buenrostro et al., 2015; Fröhlich, 2017; Hasin et al., 2017).

Transcriptomics is the study of the transcriptome that comprises the entire set of transcripts present in a cell or organism. Transcriptomics provides insight into particular types and levels of RNA molecules, including mRNA and non-coding RNA [e.g., short non-coding RNAs such as microRNA (miRNA) and long non-coding RNA], and is often used to evaluate changes in gene expression (Hasin et al., 2017). Transcriptomics approaches include hybridization-based strategies and high-throughput sequencing technologies, including DNA microarrays, RNA sequencing (RNA-seq), single cell RNA-seq (scRNA-seq), and spatial identification of transcriptomes. Hybridization-based approaches can also be used in genomics research to genotype multiple DNA regions, or in epigenomics research (e.g., ChIP-on-chip).

The proteome is the set of all expressed proteins in a cell, tissue, or organism. Proteomics research encompasses protein expression profiles, post-translational modifications, and protein networks related to cellular function, and it endeavors to understand the biological functions of proteins, which holds promise in biomarker discovery (Debnath et al., 2010). Proteomics methods involve sample purification, protein digestion, and affinity capture or sample fractionation via gel-based methods, gas chromatography (GC), or liquid chromatography (LC). The gold standard for proteomic analyses is mass spectrometry (MS)-based techniques, which enable high-throughput detection of thousands of peptides from samples to provide insights into the proteome and how it varies under particular contexts (Debnath et al., 2010; Horgan and Kenny, 2011). Metabolomics is the study of the metabolome, which is the global profile of metabolites (e.g., amino acids, lipids, sugars, and hormones) that are detectable under certain conditions. Subsets of metabolites can also be measured, such as lipids (lipidomics), ions (ionomics), and hormones (hormonomics). Metabolomics methods usually take the form of nuclear magnetic resonance (NMR) spectroscopy or MS-based approaches to identify and quantify metabolite abundance.

Data obtained from omics experiments require detailed bioinformatic analysis and statistics to organize and interpret the information. Methods used for analysis of omics datasets include enrichment and network analyses, or other statistical tools. The integration of multiple omics by interpreting data from several sources has enabled a more detailed understanding of biological processes (Culibrk et al., 2016; Karczewski and Snyder, 2018). Therefore, integrated multi-omics approaches allow researchers to delve into the molecular underpinnings of biological processes and diseases, such as healthy placental development or maldevelopment during pregnancy complications. Examples of studies that have used genomics, transcriptomics, and epigenomics approaches to study STB development are provided in Table 2. Examples of studies that have used proteomics approaches to study STB, or products produced by STB, are provided in Table 3.

**TABLE 2:** Examples of studies that used genomics, transcriptomics, and epigenomics to study STB.

Model	Method	Reference(s)	Summary
hESCs and primed hPSCs	DNA microarray	Xu et al., 2002; Sudheer et al., 2012; Chen et al., 2013; Wei et al., 2017; Tsuchida et al., 2020	Transcriptome analyses supported the notion that BMP4-treated hESCs/hPSCs differentiate into cells possessing trophoblast-like properties. BMP activation together with inhibition of both activin/nodal and FGF signaling stimulated pronounced differentiation into STB-like cells.
	WGS	Li et al., 2020	Found 6 genomic variations associated with differentiation-linked genes.
	RNA-seq	Yabe et al., 2016; Karvas et al., 2020	Characterized transcripts in hESC-derived STB, primary CTB-derived STB, and STB from early gestation placenta.
Naïve hPSCs	RNA-seq, scRNA-seq, ATAC-seq, Bisulfite-seq	Cinkornpumin et al., 2020; Dong et al., 2020; Guo et al., 2021; Io et al., 2021	Naïve hPSCs differentiate to form hTSCs with the capacity to form STBs. Naïve hPSC-derived hTSCs showed similar transcriptome signatures and chromatin accessibility to blastocyst-derived hTSCs and similar methylation patterns to placenta-derived hTSCs.
Human embryos	scRNA-seq	Lv et al., 2019; West et al., 2019	Single cell analyses identified underlying genetic networks and novel factors controlling STB development.
hTSCs	RNA-seq	Okae et al., 2018	hTSCs derived from first-trimester primary CTBs or human blastocysts can differentiate into STB. Transcriptome analyses provided evidence that hTSC-derived STB resemble primary CTB-derived STB.
CTB organoids	Bisulfite-seq, DNAm* microarray, RNA-seq	Haider et al., 2018; Turco et al., 2018	Revealed similarities between CTB organoids from first-trimester placentas and primary villus CTB and STB.
Primary CTBs	DNA microarray	Aronow et al., 2001; Rouault et al., 2016; Szilagyi et al., 2020	Classified global gene expression patterns during spontaneous differentiation into STB. Implicated new factors and genetic networks linked to signaling pathways that may be involved.
	RNA-seq	Azar et al., 2018	Coupled analysis of primary STB development to BeWo cell syncytialization and found similar genes differentially expressed during differentiation.
	ChIP-seq	Kwak et al., 2019	Illuminated global changes in binding of polymerase II and associated modified histones indicative of active or repressed chromatin during STB formation.
	miRNA microarray	Kumar et al., 2013	Identified members of the miR-17∼92 cluster and its paralog miR-106a-363 that promote CTB proliferation and were downregulated during differentiation.
	miRNA array	Ouyang et al., 2016	Characterized miRNA cargo in various STBEV subtypes.
BeWo	DNA microarray	Kudo et al., 2004; Kusama et al., 2018	Characterized transcriptome changes during forskolin-stimulated syncytialization and identified role of PKA and EPAC.
	RNA-seq	Renaud et al., 2015; Shankar et al., 2015; Zheng et al., 2017; Azar et al., 2018	Classified gene expression patterns during BeWo cell differentiation and uncovered novel genes and signaling pathways involved in this process.
	RRBS-seq	Shankar et al., 2015	Found altered CpG methylation near genes linked to STB differentiation.
	ChIP-seq	Shankar et al., 2015; Jaju Bhattad et al., 2020	Syncytialization is associated with a gain in transcriptionally active histone marks and altered histone H3 acetylation at select genomic sites.
	miRNA microarray	Dubey et al., 2018	Found that miR-92-1-5p was significantly downregulated during syncytialization.
JEG-3	DNA microarray	Msheik et al., 2019	Compared gene expression changes between JEG-3 cells and BeWo cells to identify genes potentially involved in fusion.
	RNA-seq	McConkey et al., 2016; Meinhardt et al., 2020	3D culture model of JEG-3 cells that form STB when cultured with endothelial cells. Affirmed role of YAP in promoting CTB stemness.
Placental tissue	RNA-seq	Saben et al., 2014	Transcriptome of term placenta compared to 7 other tissues revealed novel factors whose expression was localized to STB.
	scRNA-seq	Pavličev et al., 2017; Tsang et al., 2017; Liu et al., 2018; Vento-Tormo et al., 2018	Analyzed single cells from first-trimester and term placentas (and deciduas). Defined subclasses of CTBs, delineated differentiation trajectory into STB, and identified putative regulators. Inferred cell-cell interactions at the decidua-placental interface.
	DNA-seq	Poon et al., 2013; Zhang et al., 2019	No association between amount of cfDNA in maternal blood during early pregnancy and subsequent pregnancy complications. DNA isolated from placental EVs shared strong similarities with cfDNA.
	DNAm* microarray	Yuan et al., 2021	DNA methylation atlas of placental cells including laser capture dissected STB.

*DNAm, DNA methylation.

**TABLE 3:** Examples of studies that used proteomics, metabolomics, and secretomics to characterize STB function.

Model	Method	Reference(s)	Summary
hESC/hPSC models	LC-MS/MS	Sarkar et al., 2015, 2016	Analysis of cytoplasmic and nuclear proteome revealed various proteins and epigenetic regulators associated with differentiation of hESCs into trophoblast.
CTB organoids	LC-MS/MS	Turco et al., 2018	Organoids and placental villus explants produced PSGs, INSL4, hCG, KISS1, GDF15, hPL, and sorbitol.
Primary CTBs	Various types of MS	Heazell et al., 2008	Cultured CTBs and explants under different O_2_ concentrations and found 264 unique metabolites in conditioned medium and tissue lysates.
		Epiney et al., 2012	Cultured primary CTBs isolated from first-trimester placentas, term placentas, and placentas from preeclampsia, reporting 33 proteins differentially expressed between cells from healthy and preeclamptic pregnancies.
		Ouyang et al., 2016	Characterized proteins in STBEV subtypes. Exosomes were enriched for surface proteins, and apoptotic bodies and microvesicles were enriched for cytoplasmic and focal adhesion proteins.
		Salomon et al., 2013	Cultured first-trimester primary CTBs at different O_2_ tensions and characterized exosome release and composition.
Placental tissue	Various types of MS	Dunn et al., 2012	Characterized changes in metabolites between early and late-gestation placentas and between healthy and preeclamptic placentas. Differences in mitochondrial metabolites were apparent.
		Paradela et al., 2005; Zhang et al., 2010; Vandré et al., 2012	Analyzed the STB microvillus membrane and identified proteins associated with lipid raft microdomains, actin-based cytoskeletal structures, glucose transport, plasma membrane and lipid anchoring, nutrient transport, signal transduction, endo/exocytosis, and vesicular transport.
		Fisher et al., 2019	Compared changes in mitochondrial proteins between STB and CTB mitochondria.
		Burkova et al., 2019	Isolated exosomes using crude extraction and gel filtration protocols. Reported that crude extraction results in an overestimation of the number of detectable proteins.
	^1^H-NMR and LC-MS/MS	Kedia et al., 2015; Walejko et al., 2018	Characterized differences in metabolites present at the basal plate and the chorionic plate. Reported that metabolite levels altered at the basal and chorionic plates following delivery.
	iTRAQ	Shi et al., 2013	Reported differential expression of mitochondrial proteins isolated from normotensive and preeclamptic placentas.
	SOMAscan	Michelsen et al., 2019	Sampled blood from uterine and umbilical arteries and veins. Proteins with altered levels in venous blood relative to arterial blood were deduced to be secreted or absorbed by STB.
Placental explants	Various types of MS	Horgan et al., 2010	Cultured placental explants from healthy control and FGR pregnancies at different O_2_ concentrations and found 221 unique endogenous metabolites differentially produced.
		Baig et al., 2014	Identified 25 differentially expressed proteins in STB microvesicles in villus explants prepared from healthy and preeclamptic pregnancies.
		Tong et al., 2016	Characterized proteins present in macro-, micro-, and nano-sized EVs from placental explant cultures. Proteins were involved in vesicle transport, inflammation, and complement regulation.

## Omics Technologies for the Study of Early STB Development

### Using Omics to Characterize hESC and hPSC-Derived Models of Trophoblast

Omics technologies have fostered great progress toward the study of early human STB development. For instance, the use of omics has been instrumental in characterizing early hESC and hPSC models that recapitulate features of trophoblast differentiation when cultured in defined conditions. Xu et al. (2002) first demonstrated the ability of hESCs to differentiate into trophoblast-like cells via culture with BMP4. This was supported using DNA microarray to determine differentially expressed genes between BMP4-treated and untreated hESCs. By day 7 of treatment, cells expressed an array of trophoblast markers, including genes encoding hCG subunits, while transcripts associated with pluripotent cells (e.g., *OCT4*) were downregulated (Xu et al., 2002). Other studies have similarly used DNA microarray to evaluate global transcriptomic changes in BMP4-treated hESCs, consistently demonstrating gene expression patterns reminiscent of trophoblast (Marchand et al., 2011; Li et al., 2013).

Refinements in culture conditions have facilitated more detailed analyses of STB-like cells generated from hESCs. For example, Sudheer et al. (2012) used DNA microarray to show that BMP4 together with inhibition of both activin/nodal and FGF signaling stimulated pronounced differentiation into STB. Upregulated genes included those involved in hCG production, hormone biosynthesis (e.g., *CYP19A1*), and cell fusion (e.g., *ERVW-1*), whereas downregulated genes included those associated with mitosis (Sudheer et al., 2012). Yabe et al. (2016) used RNA-seq to compare hESC-derived STB isolated after 8 days of differentiation (using BMP4 and inhibitors of activin and FGF signaling) in two cell-size fractions, smaller syncytia (<40 μm) and larger syncytia (>70 μm), along with undifferentiated hESCs and primary CTBs isolated from term placenta. Notably, while the >70 μm hESC-derived STB showed a similar profile to primary CTB-derived STB, there were also major differences. For instance, several genes (e.g., *GABRP*) were highly expressed in >70 μm hESC-derived STB, but not in primary CTB-derived STB, whereas the reverse was true for others (e.g., *CSH1* as well as *PSG* and *LGALS* family members) (Yabe et al., 2016). To determine whether hESC-derived STB is more representative of STB from early or late pregnancy, Karvas et al. (2020) compared their transcriptome profile to publicly available transcriptome datasets of trophoblast from blastocysts through term. STB derived from hESCs closely resembled first-trimester STB, and transcripts expressed in hESC-derived STB that were not expressed in primary term trophoblasts (e.g., *ACTC1*, *GABRP*, *VTCN1*, and *WFDC2*) were identified as putative markers of STB in early pregnancy. Proteins encoded by these transcripts (which promote cell invasion in some cancers) showed reduced expression as gestation advanced, indicating that hESC-derived STB might represent the invasive STB population that forms soon after implantation (Karvas et al., 2020).

Sarkar et al. (2015) used proteomics methods to study the role of activin/nodal signaling during differentiation of hESCs to trophoblasts. Undifferentiated hESCs were labeled with stable isotopes and the plasma membrane and cytoplasmic fractions were compared through LC-MS/MS to fractions from cells treated for 6 days with SB431542 (an activin/nodal inhibitor). From 199 upregulated cell-surface proteins in treated cells, 83 had previously been identified in trophoblasts. KRT7 was upregulated and class I human leukocyte antigens were downregulated in SB431542-treated cells, suggesting differentiation into trophoblast (Sarkar et al., 2015). Analysis of the nuclear proteome in SB431542-treated cells showed downregulation of DNA methyltransferases such as DNMT1 while components of the BAF-A chromatin remodeling complex and SIN3 histone deacetylase (HDAC) complex were upregulated. There was also altered expression of β-catenin and CBF-1 TFs, suggesting involvement of WNT and Notch signaling in this process (Sarkar et al., 2016).

Multi-omics strategies have also been performed to study regulatory mechanisms implicated in hESC-mediated differentiation of trophoblast-like cells. Krendl et al. (2017) combined microarray, RNA-seq, scRNA-seq, ChIP-Seq, and DNA methylation analyses to profile the transcriptome and epigenome of trophoblasts derived from BMP4-treated hESCs and purified using the cell-surface marker aminopeptidase A. Furthermore, a TF circuit was identified involving GATA2, GATA3, TFAP2A, and TFAP2C that may regulate early trophoblast specification through activation of placenta-related genes and suppression of *OCT4* (Krendl et al., 2017). Liu et al. (2017a) investigated the role of *cis*-regulatory elements in this process by comparing chromatin accessibility of undifferentiated and BMP4-differentiated hESCs, which was derived from published DNase-seq datasets. This analysis was then integrated with transcriptome datasets to identify TFs binding within trophoblast-specific accessible chromatin domains (e.g., *BACH2*). The subset of these sites containing TF motifs was associated with genes controlling trophoblast invasion and placental development, and protein–protein interaction data were incorporated to construct a network highlighting candidate TFs that may be important in these processes (Liu et al., 2017a).

Similar to hESCs, transcriptome analyses of human induced PSCs (hiPSCs) cultured in defined conditions (such as with BMP4 or in micromesh culture) provide evidence that these cells possess STB-like gene expression profiles (Chen et al., 2013; Wei et al., 2017; Li et al., 2019; Mischler et al., 2021). Tsuchida et al. (2020) treated hiPSCs with BMP4 for 10 days, and cells positive for the pan-trophoblast marker KRT7 were purified and compared to undifferentiated hiPSCs by DNA microarray. Hierarchical clustering separated the two groups of cells into distinct clusters, with KRT7+ cells expressing markers representative of trophoblast lineages including STB. Furthermore, *XAGE2* and *KCNQ2*, which were upregulated in KRT7+ cells, exhibited distinct expression patterns in human placenta *in situ* (Tsuchida et al., 2020). Li et al. (2020) used whole-genome sequencing (WGS) along with published transcriptomic and epigenomic datasets to identify 6 genomic variations associated with genes upregulated following BMP4-mediated differentiation of hPSCs. One of these was a single nucleotide variation in the promoter region of *MEF2C* that increased the binding affinity of TFs to this region. This resulted in increased expression of MEF2C and its target genes, thereby promoting trophoblast differentiation (Li et al., 2020). The use of hiPSCs has also been exploited to study patient-specific STB developmental processes. For instance, hiPSCs generated from umbilical cords and exposed to culture conditions that promote trophoblast formation show stark differences in the transcriptome (as determined by RNA-seq) if the cords are collected from pregnancies with early onset preeclampsia. In particular, expression of genes associated with O_2_ responsiveness and STB formation is impaired in cells from preeclampsia, without marked changes in the DNA methylome (Sheridan et al., 2019; Horii et al., 2021).

Notably, the aforementioned studies applied hESC/hPSC differentiation paradigms that typically resulted in a heterogenous mixture of trophoblast-like cells rather than STB exclusively, with limited temporal control over differentiation events. Additionally, cells arising from these differentiation paradigms do not fulfill all criteria for trophoblast identity, suggesting either incomplete reprogramming to trophoblast or the possibility that cells other than trophoblast are formed, namely mesoderm or amnion (Bernardo et al., 2011; Roberts et al., 2014; Lee et al., 2016; Guo et al., 2021; Io et al., 2021). Nevertheless, the various omics approaches used in these studies were instrumental in characterizing these cells as having trophoblast-like properties and in discovering novel regulatory mechanisms that may be implicated in driving their differentiation. In many cases, results were validated using other trophoblast cell lines or placental tissue. Therefore, these studies have provided the groundwork for future improvements in the use of cell models for trophoblast development and advanced our understanding of early STB formation.

### Omics-Based Analysis of STB Development Using Early Human Embryos

Omics experiments making use of early human embryos provide a compelling means of investigating primitive STB development. In particular, studies utilizing human embryos have made use of scRNA-seq technology to characterize development of early trophoblast lineages, including STB, although differences in cell isolation methodologies (particularly for STB due to its multinucleated nature) could lead to variability in gene expression levels. West et al. (2019) used scRNA-seq on trophoblast cells isolated from human embryos cultured for 8-, 10-, or 12-days post-fertilization. Genes enriched in STB were consistent with its known functions (e.g., transport) in addition to pathways reflecting the nature of primitive STB during implantation (e.g., invasion). CTBs with a partial STB signature most apparent at day 10 were also identified and inferred to be mitotically active intermediate CTBs primed to fuse into STB. At day 12, this population was in decline while there was a resurgence of undifferentiated and migratory CTBs, possibly reflecting the start of villus formation (West et al., 2019). Lv et al. (2019) also used scRNA-seq to profile the transcriptome of individual trophoblast cells isolated from human embryos, but embryos were co-cultured with or without endometrial cells for 6–10 days as a model of peri-implantation development. Interestingly, genes associated with trophoblast maturation were more robustly expressed when co-cultured with endometrial stromal cells, underscoring the importance of closely mimicking the *in vivo* environment when studying early developmental events. Separate cell clusters were enriched in STB, CTB, or extravillous trophoblast (EVT) marker genes. Moreover, three time-dependent genetic networks were characterized between days 6 and 10, including an early (possibly pre-implantation) stage associated with epithelial development, a middle (peri-implantation) stage featuring expression of fusion-related genes, and a late (post-implantation) stage featuring genes associated with cell migration. The authors then determined when STB segregated from cells expressing CTB and EVT markers, demonstrating that STB first appeared in co-cultures between days 7 and 8. Putative upstream regulators were screened to identify TBX3 as a novel TF required for STB formation, which was validated through knockdown experiments using JEG-3 cells (Lv et al., 2019). Collectively, these studies provide compelling insight into the developmental dynamics of early STB.

### Using Omics to Characterize STB Development in hTSCs, Naïve hPSCs, and Organoids

Various omics approaches have also been applied to develop and profile hTSC, naïve hPSC, and organoid models that have been utilized to study STB development. For instance, Okae et al. (2018) used RNA-seq to compare CTBs and STB isolated from first-trimester placentas and identified genes predominant in each cell type. Functional annotation showed that genes overrepresented in CTBs were involved in WNT and epidermal growth factor (EGF) signal transduction pathways. It was ultimately determined that activation of WNT and EGF together with inhibition of TGF-β, HDAC, and ROCK allowed for extended culture of CTB-derived hTSCs. These hTSCs had the capacity to differentiate into STB after withdrawal of WNT and EGF signaling and exposure to forskolin. Similar cell-lines were also derived from human blastocysts. RNA-seq showed similar gene expression profiles between CTBs, EVTs, and STB generated from the stem cell-lines compared to those derived from primary first-trimester CTBs. Whole-genome bisulfite sequencing (bisulfite-seq) showed that CTB-derived and blastocyst-derived hTSCs had nearly identical DNA methylation patterns, although some differences were apparent with primary CTBs. Sequencing of miRNAs demonstrated similar global miRNA expression profiles between the three cell-types, including robust expression of miRNAs from the trophoblast-enriched chromosome 19 miRNA cluster (C19MC) (Okae et al., 2018).

While hTSCs offer exciting new ways to study STB development, generation of hTSCs through reprogramming increases access to hTSC lines from diverse genetic backgrounds, enabling integration of both normal and pathological states into the study of STB formation. Recent reports indicate that hTSCs can be derived from naïve hPSCs (reflecting pre-implantation epiblast) but not from conventional primed hPSCs (post-implantation epiblast) (Castel et al., 2020; Cinkornpumin et al., 2020; Dong et al., 2020; Guo et al., 2021; Io et al., 2021). These naïve hPSC-derived hTSCs can invariably differentiate into mature trophoblast lineages, including STB. Numerous omics methods have been used to characterize naïve hPSC-derived hTSCs. For example, Dong et al. (2020) showed through RNA-seq that naïve hTSCs had a transcriptomic signature similar to blastocyst-derived hTSCs and to that of human trophectoderm at day 12 post-fertilization, and that they were capable of differentiating into STB. ATAC-seq was also performed, revealing similar chromatin accessibility landscapes between naïve and blastocyst-derived hTSCs and identifying TEAD4 binding motifs enriched at open chromatin sites during hTSC derivation, supporting the notion that TEAD4 is important for trophoblast specification (Dong et al., 2020). Cinkornpumin et al. (2020) conducted RNA-seq coupled with whole genome bisulfite-seq comparing naïve hPSC-derived hTSCs to blastocyst- or placenta-derived hTSCs, showing similar patterns of CpG methylation between placenta-derived hTSCs and naïve hPSC-derived hTSCs, a notable exception being hypermethylation near several imprinted genes (*PEG3*, *ZFAT*, and *PROSER2-AS1*) in transdifferentiated cells that was not apparent in placenta-derived hTSCs (Cinkornpumin et al., 2020). Io et al. (2021) established culture conditions to derive trophectoderm and subsequently hTSCs with the capacity to form STB from naïve hPSCs. Comparison of their transcriptome with scRNA-seq data of human embryos affirmed the trophoblast developmental spectrum from trophectoderm to post-implantation STB formation (Io et al., 2021). Guo et al. (2021) similarly performed transcriptomic analyses (RNA-seq and scRNA-seq) which was compared to published embryo culture datasets to illustrate the differentiation trajectory from naïve hPSCs into trophectoderm following inhibition of ERK/MAPK and Nodal signaling, with capacity to differentiate further into hTSCs, CTBs, and STB (Guo et al., 2021). Collectively, these studies delineate the developmental trajectory of naïve hPSCs into trophectoderm and offer the exciting potential of investigating early STB developmental dynamics.

Using a cocktail of growth factors and inhibitors proven to facilitate organoid growth of various adult epithelial cells, Haider et al. (2018) established long-term expanding 3D CTB organoid cultures from first-trimester placentas. While organoids are capable of expansion and self-renewal *in vitro*, they also spontaneously generate functionally active, hCG-secreting STB toward the luminal surface of the organoid. RNA-seq analysis comparing CTB organoids with primary villus CTBs (freshly isolated or differentiated for 72 h into STB) was conducted, demonstrating similarities in gene expression between organoids, villus CTBs, and *in vitro*-generated STB in monolayer culture (Haider et al., 2018). Similarly, Turco et al. (2018) independently established conditions for villus trophoblast organoids, which also form STB at the luminal surface of organoids. DNA microarray and whole-genome DNA methylation analysis showed similar profiles between organoids and first-trimester placental villi, including hypomethylation of the *ELF5* promoter and expression of *GATA3*, *EGFR*, *TFAP2A*, *TFAP2C*, and C19MC miRNAs. The secretome of these organoids was also analyzed using LC-MS/MS and showed a similar production of peptides as produced by placental villus explants, including PSGs, INSL4, hCG, KISS1, GDF15, hPL, and high levels of sorbitol (Turco et al., 2018). Overall, omics methods have allowed for the development of new models and have provided extensive mechanistic insights, such as identification of possible regulatory factors, to further our understanding of early STB development.

## Omics Technologies for the Study of Villus STB Development

### Studying Villus STB Development Using DNA Microarray Transcriptomics

The emergence of high-throughput omics methods has facilitated the comprehensive study of villus STB development, which is often modeled with primary CTBs or CTB-like choriocarcinoma cells (e.g., BeWo). The majority of these studies take advantage of transcriptomics, such as DNA microarray, to better understand the molecular underpinnings governing CTB differentiation and fusion into STB. Aronow et al. (2001) used DNA microarray to analyze gene expression patterns of villus CTBs isolated from term placentas and cultured for up to 6 days, with cells cultured for 12 h as reference. Their analyses categorized differentially expressed genes into varying kinetic patterns with respect to early or late induction or repression, with most groups displaying a rapid initiation of their transcriptional pattern. This suggested that CTBs are poised to rapidly commence differentiation into STB. Genes were separated into distinct functional categories, which often showed concomitant induction and repression of genes that were tightly coupled to morphological changes (Aronow et al., 2001). Rouault et al. (2016) used DNA microarray to evaluate the transcriptome of primary villus CTBs and *in vitro* generated STB, but also included villus samples from which the CTBs were isolated. CTB-enriched genes functionally represented processes such as DNA replication and repair, while STB-enriched genes were associated with cell morphology and lipid metabolism. *In silico* analysis showed that gene networks were linked to PPARG, RXRA, and NR2F1 signaling pathways, which have been implicated in CTB differentiation. While similar functional categories were observed for CTB versus STB in comparison to results from Aronow et al. (2001), each study identified unique differentially-expressed genes, which may be partly due to the variable microarray platforms used (Rouault et al., 2016).

Kudo et al. (2004) used DNA microarray to compare global gene expression in BeWo cells over a time-course of forskolin-stimulated syncytialization. Since the onset of BeWo cell differentiation can be precisely controlled through addition of forskolin, synchronized transcriptome changes at earlier time points of differentiation can be captured in comparison to primary CTB cultures. For instance, 2 h after forskolin treatment, many more genes exhibited increased expression compared to decreased expression, although this asymmetry decreased over time. Clustering of genes into temporal expression patterns provided new insights into the dynamics of BeWo cell differentiation, with those genes transiently increased at 2 h mostly encoding TFs or cell cycle-associated proteins, and genes stimulated during later stages of differentiation predominantly encoding proteins involved in cell communication and metabolism. Other genes involved in cell adhesion and fusion had altered expression soon after forskolin exposure and prior to morphological changes (Kudo et al., 2004). Induction of BeWo cell differentiation through agents like forskolin activates two downstream molecules that both contribute to this process: PKA and exchange protein directly activated by cAMP (EPAC) (Chang et al., 2011). To identify new factors controlling syncytialization through activation of PKA or EPAC, Kusama et al. (2018) performed a DNA microarray using RNA extracted from BeWo cells stimulated using PKA- or EPAC-selective cAMP analogs, reporting far fewer transcript changes following exposure to cAMP-EPAC signaling than cAMP-PKA signaling. Two TFs upregulated following cAMP-PKA signaling (STAT5B and NR4A3) were further characterized through knockdown experiments, revealing that STAT5B contributes to STB formation while NR4A3 inhibits this process (Kusama et al., 2018).

Other studies have demonstrated that CTBs undergo both morphological differentiation (characterized by fusion of mononuclear cells) and biochemical differentiation (including production of hormones such as hCG and hPL) during syncytialization through independent mechanisms (Daoud et al., 2006, 2008). To identify genes specifically implicated in morphological differentiation, Msheik et al. (2019) performed genome-wide DNA microarray profiling comparing JEG-3 cells (which can differentiate biochemically but do not fuse in monolayer culture) with BeWo cells following forskolin treatment for 48 h. From the 32 genes that were altered in BeWo cells and not in JEG-3 cells (and thus may play roles in cell fusion), many participated in aspects of cell morphology including actin filament depolymerization, cell polarity, and protein kinase C signaling. Subsequent analyses were conducted on select genes, such as *SIK1*, which was rapidly upregulated in BeWo cells exposed to forskolin and whose silencing via CRISPR/Cas9 strongly abrogated cell fusion and, to a lesser extent, biochemical differentiation (Msheik et al., 2019). To address gaps in our understanding of villus CTB development, Szilagyi et al. (2020) employed an integrated omics approach. By consulting available gene expression data, a set of genes expressed predominantly in STB were identified. Global gene expression changes during a 7-day differentiation time-course of primary term CTBs were then evaluated using DNA microarray. By combining this data with publicly available DNase I footprinting datasets, several TFs involved in regulating differentially expressed genes (e.g., KLF10, ZNF394, and ZNF682) were identified. Moreover, the TFs were categorized into two distinct aspects of differentiation: those that governed a rapid downregulation of genes ubiquitously expressed in proliferating cells, and those involved in gradual upregulation of “placenta-specific” genes associated with STB differentiation (Szilagyi et al., 2020).

### Studying Villus STB Development Using RNA-Seq and scRNA-Seq Transcriptomics

Studies that have used transcriptomics to further our understanding of villus STB development have also made use of RNA-seq technology to quantify global gene expression data. Saben et al. (2014) used RNA-seq to identify placenta-enriched transcripts by profiling the transcriptome of term placenta compared to those from 7 other tissues including adipose, breast, and heart. While many of the top expressed genes in placental tissue were to be expected, several novel genes were detected whose expression was localized *in situ* to STB, suggesting that they may play roles in STB development or function. For example, one such gene was *DLG5*, which regulates apical polarity complexes and epithelial-to-mesenchymal transition, and thus may be important for maintaining STB integrity (Saben et al., 2014). Our group previously utilized DNA microarray coupled to RNA-seq to identify the TF OVOL1 as a key regulator of STB development. Specifically, DNA microarray comparing BeWo cells cultured under control or differentiating conditions for 24 h was performed to profile changes in gene expression, which revealed *OVOL1* as the most highly upregulated gene encoding a TF. RNA-seq was then performed comparing BeWo cells expressing control shRNA and cells deficient in OVOL1 (i.e., expressing shRNA targeting *OVOL1*) cultured under differentiating conditions. Transcripts decreased in OVOL1-deficient cells included those vital for STB endocrine function. Furthermore, knockdown of OVOL1 in BeWo cells, primary CTBs, and BMP4-treated hESCs reduced expression of several ERV genes including *ERVW-1* and *ERVFRD-1*. By examining the microarray dataset for potential intermediary targets of OVOL1, it was discovered that genes encoding key factors that maintain CTBs in a progenitor state such as *MYC*, *ID1*, and *TP63* were downregulated following induced differentiation and were subsequently shown to be direct targets of OVOL1 regulation. Therefore, OVOL1 facilitates CTB differentiation and induction of STB-associated transcripts by repressing genes that maintain progenitor traits (Renaud et al., 2015).

To further uncover genes involved in CTB differentiation, RNA-seq was utilized by Zheng et al. (2017) comparing BeWo cells treated with vehicle control or forskolin for 0, 24, and 48 h. Differentially expressed genes were associated with terms such as syncytium formation, cell fate commitment, cell junction assembly, calcium ion transport, regulation of epithelial cell differentiation, and cell morphogenesis. In particular, RNA-seq findings facilitated identification of novel candidate genes possibly involved in CTB differentiation into STB, including *CACNA1S*, *NEO1*, *MYH9*, *TNS1*, and *AMOT* (Zheng et al., 2017). Azar et al. (2018) similarly performed RNA-seq on BeWo cells before and after forskolin treatment; however, this was coupled to RNA-seq analysis of primary term CTBs before and after spontaneous syncytium formation. Although there were distinct differences, transcriptome comparison of the models revealed a large overlap in the genes differentially expressed during the CTB to STB transition, lending support to the validity of both models in reflecting aspects of syncytialization. Further interrogation of the RNA-seq datasets revealed 11 genes coordinately regulated (6 upregulated: *CGB*, *TREML2*, *CRIP2*, *PAM*, *INHA*, and *FLRG*, and 5 downregulated: *SERPINF1*, *MMP19*, *EPOP*, *KRT17*, and *SAA1*) in both models following STB formation, and whose protein expression was confirmed via western blotting and immunohistochemistry (Azar et al., 2018).

McConkey et al. (2016) used RNA-seq to support their derivation of a 3D culture model of JEG-3 cells that release hCG, fuse, and express STB-specific markers when co-cultured with human brain microvascular endothelial cells. Indeed, the 3D co-cultures shared more similar transcriptomic profiles with primary human STB than their 2D-cultured counterparts. This included genes uniquely expressed in both 3D JEG-3 cultures and primary CTB-derived STB, including *CDKN1C*, *PSG1*, and *PSG5*. Moreover, similar to primary CTB-derived STB, JEG-3 cells grown in 3D are resistant to infection by viruses and Toxoplasma gondii, which further supports the use of this co-culture system to recapitulate STB development and function (McConkey et al., 2016). Furthermore, RNA-seq performed by Meinhardt et al. (2020) affirmed the role of the Hippo signaling-associated transcriptional coactivator YAP in promoting CTB stemness and inhibiting STB differentiation. Comparing the transcriptomes of primary CTBs overexpressing constitutively active YAP with YAP-knockout JEG-3 cells (generated using CRISPR-Cas9) and non-transfected primary CTBs, it was found that constitutive YAP expression upregulated various stemness, cell cycle, and mitosis-associated genes, but repressed STB-specific transcripts. On the other hand, numerous regulators of proliferation were downregulated in the YAP knockout clones while hormones and other STB markers were elevated. Follow-up experiments illustrated that YAP-TEAD4 complexes interact with genomic regions of stemness genes to promote their induction, while also forming complexes with the histone methyltransferase EZH2 prior to binding promoter regions of STB-specific genes and silencing their expression (Meinhardt et al., 2020).

Despite technical considerations associated with isolating viable single cells from a multinucleated syncytium, scRNA-seq has been applied to the study of villus STB development. Tsang et al. (2017) used this method to identify several cell-type specific gene signatures by profiling over 24,000 cells from normal term placentas and placentas from early-onset preeclampsia. Clustering analysis enabled the delineation of the known differentiation trajectory from villus CTB toward EVT or STB, with STB further bifurcating into populations with high expression of the hormone genes *GH2* and *CGB* or high expression of fusion-related genes. This study also identified multiple genes as putative regulators of STB development (e.g., *OMG, SLC1A2*, and *ADHFE1*) (Tsang et al., 2017). Vento-Tormo et al. (2018) used scRNA-seq to map the cell-cell communication network at the human decidual-placental interface by profiling the transcriptomes of 70,000 cells from first-trimester placentas and deciduas. They developed a repository of ligand-receptor interacting pairs and a statistical tool that could predict molecular interactions between cell populations based on the cell-type specificity of the complexes. In particular, this database predicted ligand-receptor interactions that are likely to control the differentiation trajectory of trophoblast into STB, including interactions of receptors present in CTBs that are involved in cellular proliferation and differentiation (EGF2, NRP2, and MET) with their corresponding ligands expressed by other cells of the placenta, such as Hofbauer cells or placental fibroblasts (Vento-Tormo et al., 2018). Liu et al. (2018) performed scRNA-seq on sorted placental cells from first and second trimester human placentas, including CTBs and STB isolated from placental villi at 8 weeks of pregnancy (STB fragments were manually sorted using a mouth pipette based on size). Bioinformatic analyses on the gene expression profiles identified three subclasses of CTBs: a highly proliferative subtype that may be responsible for replenishing the pool of CTBs (thus serving as hTSCs), a non-proliferative subtype with high expression of adhesion genes and the gene *ERVFRD-1* that was strongly suggested to be the fusion-competent progenitor cells of the STB, and a third non-proliferative subtype that did not express *ERVFRD-1* and whose function remains unknown. Expression profiles of imprinted genes and those encoding proteins involved in DNA methylation and chromatin modification were also compared between various cell-types and shown to be divergent between CTBs and STB (e.g., high expression of *DNMT1* in CTBs that maintains DNA methylation patterns during cell replication) (Liu et al., 2018).

Pavličev et al. (2017) performed scRNA-seq on term placenta complemented with RNA-seq analysis of undifferentiated endometrial cells and decidual cells. RNA-seq analysis of laser capture-dissected STB was included to circumvent limitations associated with generating single cell populations from a multinucleated syncytium. These data were amalgamated to infer putative cell-cell interactions by assessing complementary receptor-ligand pairs across different cell-types. STB-specific genes included those associated with endocrine function (*CSH2*, *CSHL1*, *GH2*, and *CGA*), PSGs, and genes associated with immunity (*HPGD* and class II human leukocyte antigens). Interactome analysis of receptor-ligand pairs revealed abundant potential for communication between STB and decidual cells. For example, STB and decidual cells express corresponding ligand-receptor pairs for WNT family members, prostaglandins, hormones, cytokines, and growth factors that may help modulate tissue-specific functions during pregnancy (Pavličev et al., 2017). The interactome analysis was inferred through gene expression profiles, so further analysis at the protein level is needed to affirm which interactions are pertinent to pregnancy maintenance and health.

### Studying Villus STB Development Using Epigenomics Approaches

Epigenomics techniques have been used to provide more detailed insights into the dynamic epigenetic networks orchestrating villus STB development. Shankar et al. (2015) combined RNA-seq, genome-scale DNA methylation, and ChIP-seq to provide a comprehensive overview of the transcriptome and epigenome during BeWo syncytialization. RNA-seq revealed altered expression of about 3,000 genes during a 3-day forskolin treatment including *MMP9*, *SGK1*, and *TRPV2*. Global DNA methylation assessment via reduced representation bisulfite sequencing (RRBS-seq) showed altered methylation of numerous CpGs within and near genes linked to cell differentiation and commitment, with upregulated and downregulated genes showing decreased and increased methylation, respectively. Furthermore, integration of the RNA-seq dataset with genome-wide localization of key histone marks using ChIP-seq indicated that syncytialization was associated with a gain in transcriptionally active marks (H3K4me3, K9ac, K27ac, and K36me3) among genes that were either expressed constitutively or upregulated following forskolin treatment, with no change in repressive histone modifications (Shankar et al., 2015). DNA methylation has also been assessed by BeadChip microarray in cultured primary CTBs exposed to different O_2_ tensions, where it was found that low O_2_ levels stimulated hypermethylation at specific sites within the genome that potentially decrease expression of genes vital for STB formation (Yuen et al., 2013). Recently, Yuan et al. (2021) used whole genome methylation microarray to provide a cell-specific DNA methylation atlas in placental cells following fluorescence-assisted cell sorting (for most lineages); STB was isolated using enzymatic digestion because of incompatibility with fluorescence-assisted cell sorting. In STB, differential methylation at specific CpGs localized to genes more prominently expressed in this lineage (e.g., *CGA, PAPPA2*, and *CYP19A1*) compared to other placental lineages, consistent with the notion that DNA methylation serves as a key epigenetic mechanism for gene regulation in the placenta (Yuan et al., 2021).

Our group investigated the role of particular epigenetic modifications and regulators during trophoblast differentiation into STB. Using multiple CTB models, we observed reduced global histone acetylation at multiple lysine residues during STB formation. ChIP-seq analysis comparing site-specific changes in histone H3 acetylation between undifferentiated and differentiating BeWo cells showed dynamic changes in chromosomal regions such as genes associated with CTB differentiation (e.g., *TEAD4* and *OVOL1*) as well as genes with novel regulatory roles in this process (e.g., *LHX4* and *SYDE1*). These findings prompted subsequent investigations into the functional roles of specific HDAC enzymes (that catalyze histone deacetylation) during STB formation, which identified HDAC1 and HDAC2 as critical mediators driving CTB differentiation (Jaju Bhattad et al., 2020). Kwak et al. (2019) used ChIP-seq to illuminate genome-wide changes in binding of polymerase II, a crucial component of the RNA transcription machinery, as well as associated modified histones indicative of active or repressed chromatin, in primary human CTBs isolated from mid-gestation placenta before and after differentiation into STB. Examples of genes showing increased polymerase II binding in STB compared to CTBs included the cell fusion gene *ERVV-2*, *CEBPB* (encodes the TF C/EBPβ), and other genes associated with immunomodulatory functions (e.g., *PSG* family members). Genes downregulated in STB included negative regulators of differentiation (e.g., *EGR1*) and genes encoding proinflammatory TFs (e.g., *NR4A2*/*NURR1*). Moreover, while promoter enrichment of repressive histone markers remained low in STB, increased and decreased polymerase II binding to promoters of a subset of genes during differentiation was closely correlated with increased and decreased active histone marks, respectively (Kwak et al., 2019).

Knowledge into the epigenetic control of human STB development has emerged from studies that assess regulatory miRNAs genome-wide. One such study by Kumar et al. (2013) used microarray-based miRNA profiling in primary term CTBs before and after STB differentiation. Several members of the miR-17∼92 cluster and its paralog miR-106a-363 were downregulated during STB differentiation. Subsequent experiments showed that these miRNAs directly target *CYP19A1* and *GCM1* that drive STB formation, and the TF c-Myc binds to genomic regulatory regions of these miRNAs to increase their expression in proliferating CTBs, thus preventing differentiation into STB (Kumar et al., 2013). Dubey et al. (2018) analyzed miRNA expression patterns in control and forskolin-treated BeWo cells using microarray-based miRNA profiling. Among miRNAs differentially expressed during syncytialization, miR-92a-1-5p was significantly downregulated, and overexpression of this miRNA in BeWo cells inhibited cell fusion and hCG secretion. *DYSF* and *PRKACA*, genes that promote STB formation, were identified as targets for inhibition by miR-92a-1-5p (Dubey et al., 2018). Future endeavors to define the epigenetic regulatory mechanisms governing STB formation could include genome-wide identification of enhancer elements. This can be achieved via multi-omics approaches integrating assays for chromatin accessibility (e.g., ATAC-seq) with ChIP-seq identifying genomic regions containing specific enhancer marks, or with chromosome conformation capture techniques (Abdulghani et al., 2019). Whole human genome STARR-seq has recently been described and shown promise in the global quantification of enhancer activity in the human genome (Liu et al., 2017b). Additionally, multi-modal analyses capable of jointly analyzing the transcriptome along with epigenetic features (e.g., DNA methylome and chromatin accessibility) have recently been developed (Clark et al., 2018). These types of analyses would provide a more thorough understanding of the epigenome and its associations with the transcriptome during STB development.

## Using Omics to Define the STB Metabolome and Sub-Proteome

The placenta exhibits a high metabolic rate, consuming more than 40% of the O_2_ used by the entire conceptus (Bonds et al., 1986). The high metabolic rate is needed for hormone biosynthesis and metabolism as well as nutrient transport, which are key functions of the STB. Several studies have exploited MS and NMR-based technologies using placental tissue to screen for changes in the proteome and metabolome in normal pregnancies and those in various other conditions such as obesity, neural tube defects, high altitude, preeclampsia, and gestational diabetes (Van Patot et al., 2010; Chi et al., 2014; Austdal et al., 2015; Yang et al., 2015; Mary et al., 2017; Qi et al., 2017; Fattuoni et al., 2018; Sun et al., 2018; Feng et al., 2019). Using GC-MS and ultra-performance LC-MS, Dunn et al. (2012) found changes in 156 metabolites between early and late-gestation placentas, including increased levels of diglycerides, phospholipids, sphingolipids, and vitamin D-related metabolites, as well as decreased levels of triglycerides in late gestation placental tissue. An additional 86 metabolites were altered in preeclampsia, with differences particularly evident in mitochondrial metabolites and those involved in oxidative and nitrative stress. The authors caution about differences in metabolite quantities based on the position from where the sample was taken and delivery mode (cesarean versus labored), underscoring the importance of proper experimental design and rigor when performing metabolic assessments of the human placenta (Dunn et al., 2012). Furthermore, regional differences in metabolite concentrations have been measured when placental tissue samples are collected closer to the basal plate versus the chorionic plate. Specifically, using ^1^H-NMR and LC-MS/MS, elevated levels of phosphatidylcholines, choline, sphingomyelins, and several amino acids (serine, alanine, taurine, and threonine) were detected in samples collected from the basal plate, whereas higher levels of formate and very low-density lipoproteins were identified in samples collected closer to the chorionic plate (Kedia et al., 2015; Walejko et al., 2018). Notably, levels of many of these metabolites changed rapidly following delivery (Walejko et al., 2018). Given the variable levels of metabolites depending on mode of delivery, time after delivery, and position from where the sample was taken, an abundance of caution must be exercised when interpreting placental metabolomics. Notably, these studies were conducted using whole placental tissue, so the contribution of STB to these findings is uncertain, especially given reports that CTBs are more metabolically active than STB (Kolahi et al., 2017).

To circumvent some of the variability associated with sampling, labor, and (to some extent) cellular heterogeneity of placental tissue, researchers have analyzed endogenous and secreted metabolites from placental explants and CTBs cultured for defined periods of time. For example, Heazell et al. (2008) prepared explants from term uncomplicated pregnancies and cultured them in different O_2_ atmospheres for up to 96 h. Subsequently, conditioned medium and tissue lysates were analyzed using GC-MS, where 264 unique metabolite peaks were detected and 2-deoxyribose, threitol, and erythritol were elevated in explants cultured in 1% O_2_ relative to 20% O_2_ (Heazell et al., 2008). Horgan et al. (2010) used a similar strategy to determine whether O_2_-dependent metabolic changes were evident in placental explants prepared from FGR pregnancies. They reported 1,676 metabolite features, and 221 unique endogenous metabolites differentially detected between placental explants prepared from healthy control and FGR pregnancies, notably those involved in glycerophospholipid and tryptophan metabolism (Horgan et al., 2010). Functional proteomic analyses have also been performed with primary CTBs after variable periods of culture following isolation from placentas and BeWo cells following forskolin treatment, although typically the extent of syncytialization is not reported (Hoang et al., 2001; Nampoothiri et al., 2007; Sun et al., 2007; Johnstone et al., 2011).

Sub-proteomics analyses have been used to characterize components of the STB microvillus membrane and mitochondria. Paradela et al. (2005) performed detergent-free LC-MS/MS to evaluate the composition of the STB microvillus membrane, identifying 57 proteins primarily associated with lipid raft microdomains such as annexins (ANXA1, ANXA2) and placental alkaline phosphatase, as well as proteins involved in actin-based cytoskeletal structures (CLIC5) and glucose transport (GLUT1) (Paradela et al., 2005). Zhang et al. (2010) used a similar detergent-free enrichment of STB microvillus membranes along with polyacrylamide gel electrophoresis, in-gel trypsin digestion, and nano-LC-MS/MS. They identified 292 proteins, including 161 proteins that are associated with the plasma membrane and are involved in lipid anchoring, nutrient transport, signal transduction, and vesicular trafficking (Zhang et al., 2010). Vandré et al. (2012) used cationic colloidal silica particles to isolate enriched preparations of microvilli containing the apical plasma membrane from placentas, and then performed LC-nanospray MS/MS. They identified 340 non-redundant proteins associated with pathways such as endocytosis, exocyst complex, and exocytosis, as well as those involved in vesicular transport such as flotillin-1, dysferlin, and myoferlin. Out of these 340 proteins, 208 were not previously detected in the studies conducted by Paradela et al. (2005) or Zhang et al. (2010), emphasizing the variability of this approach based on tissue sampling, extraction approaches, and analyses (Vandré et al., 2012).

Fisher et al. (2019) used sequential centrifugation to enrich mitochondrial fractions based on the distinct structural differences apparent between mitochondria in CTBs (larger mitochondria with defined cristae) and STB (smaller and punctate mitochondria with diffuse cristae). LC-MS/MS was then performed to evaluate proteomic differences between STB and CTB mitochondria. There were 24 proteins decreased and 5 proteins increased in STB mitochondria compared to CTB mitochondria (Fisher et al., 2019). Differences were validated using western blotting, and in many cases, were consistent with cell-type specific differences at the transcript level as determined by cross-referencing publicly available scRNA-seq datasets (Pavličev et al., 2017; Suryawanshi et al., 2018). Many of the proteins decreased in STB mitochondria are involved in key stages of electron transport complex assembly as well as carbohydrate, fatty acid, and amino acid metabolism, which is consistent with the notion that STB may be less metabolically active than CTBs. Proteomic analyses using iTRAQ labeling have also revealed differences in the levels of 26 mitochondrial proteins from placentas of preeclamptic pregnancies relative to normotensive controls, including those involved in fatty acid oxidation, reactive O_2_ species generation, and the tricarboxylic acid cycle. Although this analysis was conducted using whole placental tissue, immunostaining subsequently localized selected mitochondrial proteins to STB, including TFRC, PRDX3, and HSPE1 (Shi et al., 2013).

## Using Omics to Define the STB Secretome

### Proteomic Characterization of Factors Released by STB

Syncytiotrophoblast releases numerous hormones and other substances into maternal (and possibly fetal) blood. Omics technologies have provided deeper insight into the plethora of substances produced and released by STB. Epiney et al. (2012) analyzed conditioned media from CTBs cultured up to 72 h that were isolated from first-trimester placentas, term placentas, or placentas from preeclampsia using LC-MS/MS. They found 164 proteins in pooled supernatants, including 33 proteins that were differentially expressed between cells from healthy and preeclamptic pregnancies. Notably, higher levels of coagulation factor XIII were detected in control relative to preeclamptic samples, and levels were undetectable in first-trimester CTB samples. PSGs, CG-β, apolipoproteins (APOE), and the actin binding protein transgelin-2 (TAGLN2) also showed altered expression in samples from preeclamptic placentas (Epiney et al., 2012).

Michelsen et al. (2019) sampled blood in 35 pregnant women from four vessels: two maternal (radial artery and uterine vein) and two fetal (umbilical artery and vein). Slow off-rate modified aptamer (SOMAscan) technology was then used to determine levels of 1,310 proteins in maternal and fetal arterial and venous blood. Proteins that were increased in venous blood relative to arterial blood were surmised to be secreted by the placenta into the maternal or fetal circulation. There were 34 proteins secreted into the maternal circulation presumably by STB, including hormones and growth factors (PlGF, GDF15, FGF1, INHBA, and IGFBP7), annexins (ANXA1 and ANXA2), WNT signaling antagonists (DRP1 and DRP4), and chemokines (CXCL10). Increased levels of many of these proteins were identified as gestation progressed, including PlGF, GDF15, IGFBP7, and INHBA. Nine proteins exhibited decreased levels in uterine vein blood, including VEGF, APOB, and parathyroid hormone, suggesting that these proteins bind to STB and undergo further processing or degradation by the placenta. There were also 341 proteins with higher levels in umbilical vein blood compared to umbilical artery blood, indicating that the placenta may secrete these factors directly into fetal blood, although the cell-type responsible for producing these proteins is not yet clear. Although SOMAscan technology is limited to analyzing levels of 1,310 proteins out of the more than 20,000 in the human proteome, it provides insight into the abundance of proteins secreted by the placenta (Michelsen et al., 2019).

### Proteomic Analysis of STBEVs

STBEVs are released by STB into maternal circulation and have the potential to provide important diagnostic information about placental health. Therefore, studies have used omics strategies to characterize the composition of STBEVs. Baig et al. (2014) prepared villus explants from healthy term placentas and placentas from preeclamptic women and then isolated microvesicles from conditioned media for gel electrophoresis, in-gel digestion, and LC-MS/MS after 72 h culture. The authors identified 421 proteins within STB microvesicles. There were 25 proteins differentially expressed in STB microvesicles between normal and preeclamptic women, including increased levels of annexins (ANXA2 and ANXA4), heat shock proteins (HSPB, HSPA8, and HSPA5), and cytoskeletal proteins (ACTB, ACTN1, TUBBA1C, TUBB4B, and TUBB) in preeclampsia, as well as decreased levels of integrins (ITGAV and ITGB1), complement regulatory proteins, and some histones (which may be due to defective DNA repair or increased DNA damage). Functional pathway analysis on the differentially expressed proteins revealed terms such as cell death and survival, cellular assembly and organization, immune response, lipid metabolism, endothelial dysfunction, and intercellular junctions (Baig et al., 2014).

Ouyang et al. (2016) isolated apoptotic bodies, microvesicles, and exosomes from primary CTBs (cultured up to 72 h) and used LC-electrospray ionization-MS/MS and miRNA Taqman card PCR to characterize phospholipids, proteins, and miRNA cargo in these STBEV subtypes. Phospholipidomic analysis revealed 11 major classes of phospholipids within STBEVs, with a notably higher content of phospholipids that promote membrane stability (e.g., phosphatidylcholine) in exosomes compared to apoptotic bodies and microvesicles. Proteomic analysis identified 1,684 proteins in STBEVs. In general, exosomes were enriched for surface proteins expressed by other cell-types, including tetraspanins (e.g., CD9 and CD63), syndecan-1, syntenin-1, integrins, and endosomal complex proteins (e.g., CHMP2A and CHMP3), but also contained proteins that are predominantly expressed in the placenta, including CD276, placental alkaline phosphatase, and the fusogens syncytin-1 and syncytin-2. Apoptotic bodies and microvesicles primarily contained cytoplasmic and focal adhesion proteins such as MYH9/10, PLEC, and TLN1. Interestingly, all three types of EVs contained similar profiles of miRNAs that are distinct from miRNAs in non-vesicular form. Notably, STBEVs contained abundant C19MC miRNAs, which confer immunomodulatory properties to non-placental cells including resistance to a broad range of viruses (Delorme-Axford et al., 2013; Ouyang et al., 2016). STBEVs also modulate inflammatory cytokine production from peripheral blood mononuclear cells (Southcombe et al., 2011), and contain a variety of cytokines and growth factors either on their surface (e.g., CRP, IL-6, and IL-8) or internally (e.g., CCL5, TGF-β, IL-10, IL-33, CXCL10, MIF, and TRAIL), further supporting an immunoregulatory function of STBEVs (Fitzgerald et al., 2018).

Tong et al. (2016) performed proteomic analysis of macro, micro, and nano-sized EVs from placental explant cultures collected from 56 first-trimester placentas. Macro, micro, and nano-sized EVs were collected and characterized by LC-MS/MS, and 1,585, 1,656, and 1,476 proteins were identified in each, respectively. Many (1125) of these proteins were detectable in all three fractions of EVs. Gene ontology pathway analysis revealed enrichment of proteins involved in vesicle transport/internalization and inflammation, including ANXA5, CALR, CD31, CD47, RPS4, SERPINE1 (also known as PAI1), as well as proteins implicated in complement regulation (C3, MCP, DAF, and protectin) (Tong et al., 2016). Salomon et al. (2013) assessed the impact of different O_2_ tensions (8, 3, or 1% O_2_) on exosome release and composition in first-trimester CTBs. In total, over 160 exosomal proteins were detected using LC-MS/MS. Lower O_2_ tensions were associated with increased levels of EV-associated proteins, particularly those associated with hypoxia and IL-8 signaling. Other exosomal proteins were related to cellular movement (e.g., MMP-9, TGF-β, MAPK, and VEGF) (Salomon et al., 2013).

Burkova et al. (2019) isolated exosomes from term placental extracts using a crude extraction method with and without an additional gel filtration protocol. The authors report that crude extraction leads to contamination with additional proteins, whereas gel filtration appears to avoid impurities. MALDI-MS/MS analysis of filtrated placental exosomes revealed expression of 12 proteins: tetraspanins (CD81 and CD63), annexins (ANXA1, ANXA2, and ANXA5), cytoplasmic proteins (actin, ACTN4), placental alkaline phosphatase, and serum proteins (transferrin, hemoglobin subunits, albumin, and immunoglobulins). The total number of detectable proteins was lower when compared to the number of proteins identified in other studies. It was suggested that crude extraction of proteins from exosomes can result in an overestimation of the number of detectable proteins, underscoring the importance of effective exosome extraction protocols and rigorous protein characterization (Burkova et al., 2019).

One limitation in STBEV omics research has been the variable extraction protocols resulting in differing findings about the number and identity of proteins detected in STBEVs. In order to identify consistencies among the various studies, Familari et al. (2017) performed a meta-analysis of 6 proteomic datasets from trophoblast-derived EVs. Only 3 proteins were identified in common with all 6 datasets: albumin, FN1, and PAI1. An additional 4 proteins – C3, hemoglobin delta, transferrin, and THBS1 – were identified in 5 of the 6 datasets. Notably, datasets used in this meta-analysis were not exclusively restricted to STB (for instance, immortalized EVT cell-lines were used in some studies), so the lack of consistency between studies may be due in part to the cell models selected for inclusion in the meta-analyses. Different methodologies for exosome extraction and proteomic approaches may also contribute to variability between studies (Familari et al., 2017).

## Omics Approaches Used to Characterize STB as a Source of cfDNA

Syncytiotrophoblast is a major source of cfDNA in maternal blood, accounting for 5–10% of total circulating DNA in maternal serum (reviewed in Hahn and Holzgreve, 2002; Hudecova et al., 2014). DNA sequencing (DNA-seq) of cfDNA has revolutionized non-invasive prenatal diagnostic testing and is widely used to detect *de novo* mutations and common fetal aneuploidies like trisomy 21 (Bianchi, 2019; Guy et al., 2019). Intriguingly, the quantity of cfDNA is fivefold higher in the blood of women with preeclampsia compared to healthy control women (Lo et al., 1999; Rafaeli-Yehudai et al., 2018). However, Poon et al. (2013) suggest that cfDNA quantity may in fact not be useful as an early predictive marker of adverse pregnancy outcomes. These authors isolated maternal plasma from 1,949 women at 11–13 weeks of gestation and conducted DNA-seq to determine the proportion of fetal and maternal cfDNA. Using this strategy, it was determined that the proportion of fetal to maternal cfDNA differed based on certain features in the patient population (e.g., ethnicity and smoking habits), but there was no association between the amount of fetal or maternal cfDNA during early pregnancy and subsequent development of a pregnancy complication (Poon et al., 2013). Furthermore, Zhang et al. (2019) demonstrated through DNA-seq that DNA isolated from placental EVs shared strong similarities with cfDNA. Given the superior stability of DNA encapsulated in lipid bilayers, EV-associated DNA may have potential clinical value for non-invasive prenatal testing of less common fetal aneuploidies or DNA modifications (Zhang et al., 2019).

A potential limitation when considering the clinical application of STB-derived DNA as a diagnostic indicator of pregnancy health is the confined placental mosaicism and chromosomal aberrations in trophoblast-derived tissue that may not be representative of the fetal genetic landscape (Peñaherrera et al., 2012; Kasak et al., 2015). Coorens et al. (2021) used whole-genome sequencing of bulk placental samples and laser capture dissected STB and found significant somatic mutagenesis that rivals childhood cancers in terms of base substitutions, copy number variation and overall mutagenic burden. Comparatively, non-trophoblast tissues (umbilical cord and villus core) did not possess frequent mutations or chromosomal irregularities. Despite the drastic alterations to the STB genome, the pregnancies themselves were seemingly normal with no obvious placental pathology or adverse pregnancy outcome (Coorens et al., 2021). Further studies are required to determine whether aberrations in the STB genome have functional consequences, and whether frequent mutagenesis of the STB genome will complicate its potential diagnostic utility to gauge pregnancy health.

## Conclusions and Perspectives

**FIGURE 3:**
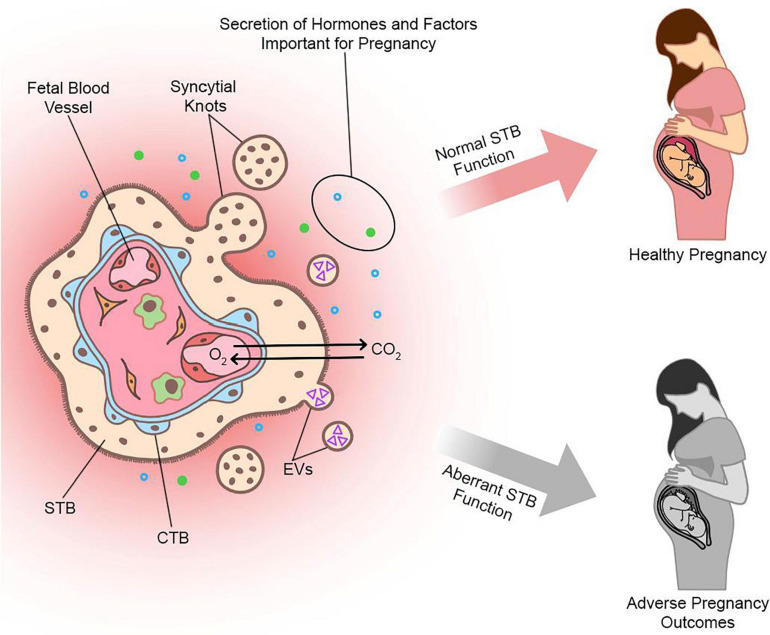
Schematic illustrating the multifunctional importance of STB for healthy pregnancy. A cross section of a chorionic villus near term is shown. O_2_, micronutrients, immunoglobulins, water, and various other substances pass across the STB layer to gain access to blood vessels in the villus core, through which they can be carried to the fetus. CO_2_ and waste products diffuse across the STB layer from fetal to maternal blood. STB also produces and secretes a variety of factors into maternal blood, including peptide and steroid hormones, growth factors, EVs, and larger vesicles (e.g., syncytial knots). Given the diverse functions of STB for pregnancy success and its contiguity with maternal blood, aberrant STB function can contribute to adverse pregnancy outcomes.

The use of omics-based approaches has provided unprecedented insights into the formation and function of STB. Where relevant, examples of how omics technologies have characterized changes in STB function in disease states were discussed (Figure 3). Several recent reviews, although not focused specifically on STB, have summarized the use of omics approaches in pregnancy complications. The reader is directed to these articles for a comprehensive discussion of omics approaches in pregnancy disease states (Herrera-Van Oostdam et al., 2019; Benny et al., 2020; Yong and Chan, 2020).

In assembling this review, several challenges became apparent that should be considered in future studies incorporating omics approaches to understand STB biology. One of these challenges is the lack of consistency between different studies. While it is not possible to mitigate all nuances between studies, consistency can be enhanced by including a detailed description of patient populations and sampling procedures (if applicable), rigorous analysis, validation with multiple techniques or models using independent samples, comprehensive discussion of limitations, and objective interpretation of results. The possibility of using a multi-omics approach is gaining traction to circumvent some of the limitations associated with a “one omics” strategy. For example, Turco et al. (2018) used DNA microarray, DNA-seq following bisulfite conversion, and LC-MS/MS approaches to characterize placental organoids developed in their lab, and Than et al. (2018) used transcriptomic and proteomic data from multiple publicly-available datasets combined with hypothesis-driven molecular approaches to reveal novel pathways associated with preeclampsia. Simultaneous measurements of two or more modalities on the same sample have also been described, which empower a more comprehensive interrogation of cell and tissue function (Zhu et al., 2020). The use of multi-omics approaches would also be greatly facilitated by better raw data accessibility, sharing, collaboration, and harmonization among the research community, potentially enabling more consistency across multiple studies.

Another challenge in understanding STB biology is the limited availability of cell and tissue models. Placental biopsies following delivery are frequently used to evaluate changes in placental function in normal and diseased states, but in many cases the specific role of STB is uncertain because biopsies contain many cell-types in addition to STB. Moreover, samples may vary even if collected from different regions of the same placenta (Konwar et al., 2019). This variation may contribute to differences in findings from study groups and reinforces the importance of consistent sampling procedures and cautious interpretation of data when inferring STB-specific functions from tissue. Cell, organoid, and explant models are available to study STB differentiation, but they all possess some limitations including heterogeneity (for example, not all primary CTBs form large syncytia in culture). In most cases, the complex 3D anatomical configuration along with the dynamic endocrinological, immunological, and hemodynamic environment characteristic of the *in vivo* milieu is not reflected in culture models. For instance, stark differences in trophoblast gene expression profiles are apparent when co-cultured with decidualized stromal cells compared to culture without these cells, highlighting the importance of modeling the *in vivo* environment as closely as possible (Lv et al., 2019).

Another limitation in the study of STB is its characteristic morphology and fragility that complicates cell isolation for *ex vivo* analyses. It is not yet possible to isolate and culture intact, purified STB from placentas, explants, or organoids to study STB in isolation or for use in functional studies. Although the recent upsurge in single cell omics has revolutionized the capacity to study specific cell lineages in complex tissue, STB poses a unique challenge for preparing single cells for analyses because of its enlarged, multinucleated nature. Future exploration incorporating single nucleus RNA-seq may enable a more robust characterization of STB at various stages of its life cycle, whereas spatial transcriptomics offers a promising avenue to study STB biology while preserving its native architectural integrity.

Omics technologies are advancing at a startling rate and are poised to continue providing unparalleled insights into STB development and function. Integration of multiple omics strategies in combination with hypothesis-driven mechanistic approaches and improvements in available cell models will enable a better understanding of how this cell lineage functions in normal and diseased states. Given the importance of STB for pregnancy health and its unique position contiguous with maternal blood, studies of STB biology that incorporate omics approaches will be instrumental for driving discovery of novel biomarkers of placental stress or pregnancy disease.

## Author Contributions

AJ, MJ, and SR wrote and edited the manuscript. GJB edited the manuscript and contributed to preparation of the tables. All authors approved the final version of the manuscript.

## Conflict of Interest

The authors declare that the research was conducted in the absence of any commercial or financial relationships that could be construed as a potential conflict of interest.

## References

[B1] Abdulghani M. Jain A. Tuteja G. (2019). Genome-wide identification of enhancer elements in the placenta. *Placenta* 79 72–77. 10.1016/j.placenta.2018.09.003 30268337 PMC6420402

[B2] Amita M. Adachi K. Alexenko A. P. Sinha S. Schust D. J. Schulz L. C. (2013). Complete and unidirectional conversion of human embryonic stem cells to trophoblast by BMP4. *Proc. Natl. Acad. Sci. U.S.A* 110 E1212–E1221. 10.1073/pnas.1303094110 23493551 PMC3612666

[B3] Aronow B. J. Richardson B. D. Hangwerger S. (2001). Microarray analysis of trophoblast differentiation: gene expression reprogramming in key gene function categories. *Physiol. Genomics* 6 105–116. 10.1152/physiolgenomics.2001.6.2.105 11459926

[B4] Austdal M. Thomsen L. C. V. Tangerås L. H. Skei B. Mathew S. Bjørge L. (2015). Metabolic profiles of placenta in preeclampsia using HR-MAS MRS metabolomics. *Placenta* 36 1455–1462. 10.1016/j.placenta.2015.10.019 26582504

[B5] Azar C. Valentine M. Trausch-Azar J. Druley T. Nelson D. M. Schwartz A. L. (2018). RNA-Seq identifies genes whose proteins are transformative in the differentiation of cytotrophoblast to syncytiotrophoblast, in human primary villous and BeWo trophoblasts. *Sci. Rep.* 8:5142. 10.1038/s41598-018-23379-2 29572450 PMC5865118

[B6] Baczyk D. Dunk C. Huppertz B. Maxwell C. Reister F. Giannoulias D. (2006). Bi-potential behaviour of cytotrophoblasts in first trimester chorionic villi. *Placenta* 27 367–374. 10.1016/j.placenta.2005.03.006 15950280

[B7] Baig S. Kothandaraman N. Manikandan J. Rong L. Ee K. Hill J. (2014). Proteomic analysis of human placental syncytiotrophoblast microvesicles in preeclampsia. *Clin. Proteomics* 11:40. 10.1186/1559-0275-11-40 25469110 PMC4247627

[B8] Baines K. J. Renaud S. J. (2017). Transcription factors that regulate trophoblast development and function. *Prog. Mol. Biol. Transl. Sci.* 145 39–88. 10.1016/bs.pmbts.2016.12.003 28110754

[B9] Benny P. A. Alakwaa F. M. Schlueter R. J. Lassiter C. B. Garmire L. X. (2020). A review of omics approaches to study preeclampsia. *Placenta* 92 17–27. 10.1016/j.placenta.2020.01.008 32056783 PMC7306500

[B10] Bernardo A. S. Faial T. Gardner L. Niakan K. K. Ortmann D. Senner C. E. (2011). BRACHYURY and CDX2 mediate BMP-induced differentiation of human and mouse pluripotent stem cells into embryonic and extraembryonic lineages. *Cell Stem Cell* 9 144–155. 10.1016/j.stem.2011.06.015 21816365 PMC3567433

[B11] Bianchi D. W. (2019). Turner syndrome: New insights from prenatal genomics and transcriptomics. *Am. J. Med. Genet. Part C Semin. Med. Genet.* 181 29–33. 10.1002/ajmg.c.31675 30706680 PMC10110351

[B12] Bonds D. R. Crosby L. O. Cheek T. G. Hägerdal M. Gutsche B. B. Gabbe S. G. (1986). Estimation of human fetal-placental unit metabolic rate by application of the Bohr principle. *J. Dev. Physiol.* 8 49–54. 3082967

[B13] Borges M. Bose P. Frank H. G. Kaufmann P. Pötgens A. J. G. (2003). A two-colour fluorescence assay for the measurement of syncytial fusion between trophoblast-derived cell lines. *Placenta* 24 959–964. 10.1016/S0143-4004(03)00173-5 14580378

[B14] Buenrostro J. D. Wu B. Chang H. Y. Greenleaf W. J. (2015). ATAC-seq: A method for assaying chromatin accessibility genome-wide. *Curr. Protoc. Mol. Biol.* 2015 21.29.1–21.29.9. 10.1002/0471142727.mb2129s109 25559105 PMC4374986

[B15] Burkova E. E. Grigor’eva A. E. Bulgakov D. V. Dmitrenok P. S. Vlassov V. V. Ryabchikova E. I. (2019). Extra purified exosomes from human placenta contain an unpredictable small number of different major proteins. *Int. J. Mol. Sci.* 20:2434. 10.3390/ijms20102434 31100946 PMC6566543

[B16] Burton G. J. (2011). Deportation of syncytial sprouts from the term human placenta. *Placenta* 32 96–98. 10.1016/j.placenta.2010.09.015 20950856

[B17] Burton G. J. Jauniaux E. (2018). Pathophysiology of placental-derived fetal growth restriction. *Am. J. Obstet. Gynecol.* 218 S745–S761. 10.1016/j.ajog.2017.11.577 29422210

[B18] Burton G. J. Jones C. J. P. (2009). Syncytial knots, sprouts, apoptosis, and trophoblast deportation from the human placenta. *Taiwan. J. Obstet. Gynecol.* 48 28–37. 10.1016/S1028-4559(09)60032-2 19346189

[B19] Castel G. Meistermann D. Bretin B. Firmin J. Blin J. Loubersac S. (2020). Induction of human trophoblast stem cells from somatic cells and pluripotent stem cells. *Cell Rep.* 33:108419. 10.1016/j.celrep.2020.108419 33238118

[B20] Chamley L. W. Chen Q. Ding J. Stone P. R. Abumaree M. (2011). Trophoblast deportation: Just a waste disposal system or antigen sharing? *J. Reprod. Immunol.* 88 99–105. 10.1016/j.jri.2011.01.002 21334749

[B21] Chang C.-W. Chang G.-D. Chen H. (2011). A novel cyclic AMP/Epac1/CaMKI signaling cascade promotes GCM1 desumoylation and placental cell fusion. *Mol. Cell. Biol.* 31 3820–3831. 10.1128/mcb.05582-11 21791615 PMC3165715

[B22] Chen Y. Wang K. Chandramouli G. V. R. Knott J. G. Leach R. (2013). Trophoblast lineage cells derived from human induced pluripotent stem cells. *Biochem. Biophys. Res. Commun.* 436 677–684. 10.1016/j.bbrc.2013.06.016 23774580 PMC3750115

[B23] Chi Y. Pei L. Chen G. Song X. Zhao A. Chen T. (2014). Metabonomic profiling of human placentas reveals different metabolic patterns among subtypes of neural tube defects. *J. Proteome Res.* 13 934–945. 10.1021/pr4009805 24397701

[B24] Cinkornpumin J. K. Kwon S. Y. Guo Y. Hossain I. Sirois J. Russett C. S. (2020). Naive human embryonic stem cells can give rise to cells with a trophoblast-like transcriptome and methylome. *Stem Cell Rep.* 15 198–213. 10.1016/j.stemcr.2020.06.003 32619492 PMC7363941

[B25] Clark S. J. Argelaguet R. Kapourani C. A. Stubbs T. M. Lee H. J. Alda-Catalinas C. (2018). ScNMT-seq enables joint profiling of chromatin accessibility DNA methylation and transcription in single cells. *Nat. Commun.* 9:781. 10.1038/s41467-018-03149-4 29472610 PMC5823944

[B26] Coorens T. H. H. Oliver T. R. W. Sanghvi R. Sovio U. Cook E. Vento-Tormo R. (2021). Inherent mosaicism and extensive mutation of human placentas. *Nature* 592 80–85. 10.1038/s41586-021-03345-1 33692543 PMC7611644

[B27] Culibrk L. Croft C. A. Tebbutt S. J. (2016). Systems biology approaches for host-fungal interactions: an expanding multi-omics frontier. *Omi. A J. Integr. Biol.* 20 127–138. 10.1089/omi.2015.0185 26885725 PMC4799697

[B28] Daoud G. Le bellego F. Lafond J. (2008). PP2 regulates human trophoblast cells differentiation by activating p38 and ERK1/2 and inhibiting FAK activation. *Placenta* 29 862–870. 10.1016/j.placenta.2008.07.011 18783823

[B29] Daoud G. Rassart É Masse A. Lafond J. (2006). Src family kinases play multiple roles in differentiation of trophoblasts from human term placenta. *J. Physiol.* 571 537–553. 10.1113/jphysiol.2005.102285 16410281 PMC1805791

[B30] Debnath M. Prasad G. B. K. S. Bisen P. S. (2010). *Molecular Diagnostics: Promises and Possibilities.* Dordrecht: Springer Netherlands, 1–520. 10.1007/978-90-481-3261-4

[B31] Delorme-Axford E. Donker R. B. Mouillet J. F. Chu T. Bayer A. Ouyang Y. (2013). Human placental trophoblasts confer viral resistance to recipient cells. *Proc. Natl. Acad. Sci. U.S.A.* 110 12048–12053. 10.1073/pnas.1304718110 23818581 PMC3718097

[B32] Dong C. Beltcheva M. Gontarz P. Zhang B. Popli P. Fischer L. A. (2020). Derivation of trophoblast stem cells from naïve human pluripotent stem cells. *Elife* 9:e52504. 10.7554/eLife.52504 32048992 PMC7062471

[B33] Dubey R. Malhotra S. S. Gupta S. K. (2018). Forskolin-mediated BeWo cell fusion involves down-regulation of miR-92a-1-5p that targets dysferlin and protein kinase cAMP-activated catalytic subunit alpha. *Am. J. Reprod. Immunol.* 79:e12834. 10.1111/aji.12834 29484758

[B34] Dunn W. B. Brown M. Worton S. A. Davies K. Jones R. L. Kell D. B. (2012). The metabolome of human placental tissue: investigation of first trimester tissue and changes related to preeclampsia in late pregnancy. *Metabolomics* 8 579–597. 10.1007/s11306-011-0348-6

[B35] Epiney M. Ribaux P. Arboit P. Irion O. Cohen M. (2012). Comparative analysis of secreted proteins from normal and preeclamptic trophoblastic cells using proteomic approaches. *J. Proteomics* 75 1771–1777. 10.1016/j.jprot.2011.12.021 22234358

[B36] Familari M. Cronqvist T. Masoumi Z. Hansson S. R. (2017). Placenta-derived extracellular vesicles: their cargo and possible functions. *Reprod. Fertil. Dev.* 29 433–447. 10.1071/RD15143 26411402

[B37] Fattuoni C. Mandò C. Palmas F. Anelli G. M. Novielli C. Parejo Laudicina E. (2018). Preliminary metabolomics analysis of placenta in maternal obesity. *Placenta* 61 89–95. 10.1016/j.placenta.2017.11.014 29277276

[B38] Feng Y. He Y. Wang J. Yuan H. Zou J. Yang L. (2019). Application of iTRAQ proteomics in identification of the differentially expressed proteins of placenta of pregnancy with preeclampsia. *J. Cell. Biochem.* 120 5409–5416. 10.1002/jcb.27819 30506707

[B39] Fisher J. J. McKeating D. R. Cuffe J. S. Bianco-Miotto T. Holland O. J. Perkins A. V. (2019). Proteomic analysis of placental mitochondria following trophoblast differentiation. *Front. Physiol.* 10:1536. 10.3389/fphys.2019.01536 31920727 PMC6933824

[B40] Fitzgerald W. Gomez-Lopez N. Erez O. Romero R. Margolis L. (2018). Extracellular vesicles generated by placental tissues ex vivo: a transport system for immune mediators and growth factors. *Am. J. Reprod. Immunol.* 80:e12860. 10.1111/aji.12860 29726582 PMC6021205

[B41] Fogarty N. M. E. Ferguson-Smith A. C. Burton G. J. (2013). Syncytial knots (Tenney-parker changes) in the human placenta: evidence of loss of transcriptional activity and oxidative damage. *Am. J. Pathol.* 183 144–152. 10.1016/j.ajpath.2013.03.016 23680657

[B42] Fröhlich E. (2017). Role of omics techniques in the toxicity testing of nanoparticles. *J. Nanobiotechnol.* 15:84. 10.1186/s12951-017-0320-3 29157261 PMC5697164

[B43] Gauster M. Moser G. Orendi K. Huppertz B. (2009). Factors involved in regulating trophoblast fusion: potential role in the development of preeclampsia. *Placenta* 30 49–54. 10.1016/j.placenta.2008.10.011 19027159

[B44] Gerbaud P. Taskén K. Pidoux G. (2015). Spatiotemporal regulation of cAMP signaling controls the human trophoblast fusion. *Front. Pharmacol.* 6:202. 10.3389/fphar.2015.00202 26441659 PMC4569887

[B45] Guo G. Stirparo G. G. Strawbridge S. E. Spindlow D. Yang J. Clarke J. (2021). Human naive epiblast cells possess unrestricted lineage potential. *Cell Stem Cell* 28, 1040–1056. 10.1016/j.stem.2021.02.025 33831366 PMC8189439

[B46] Guy C. Haji-Sheikhi F. Rowland C. M. Anderson B. Owen R. Lacbawan F. L. (2019). Prenatal cell-free DNA screening for fetal aneuploidy in pregnant women at average or high risk: results from a large US clinical laboratory. *Mol. Genet. Genomic Med.* 7:e545. 10.1002/mgg3.545 30706702 PMC6418367

[B47] Hahn S. Holzgreve W. (2002). Fetal cells and cell-free fetal DNA in maternal blood: New insights into pre-eclampsia. *Hum. Reprod. Update* 8 501–508. 10.1093/humupd/8.6.501 12498420

[B48] Haider S. Meinhardt G. Saleh L. Kunihs V. Gamperl M. Kaindl U. (2018). Self-renewing trophoblast organoids recapitulate the developmental program of the early human placenta. *Stem Cell Rep.* 11 537–551. 10.1016/j.stemcr.2018.07.004 30078556 PMC6092984

[B49] Hasin Y. Seldin M. Lusis A. (2017). Multi-omics approaches to disease. *Genome Biol.* 18 1–15. 10.1186/s13059-017-1215-1 28476144 PMC5418815

[B50] Heazell A. E. P. Brown M. Dunn W. B. Worton S. A. Crocker I. P. Baker P. N. (2008). Analysis of the metabolic footprint and tissue metabolome of placental villous explants cultured at different oxygen tensions reveals novel redox biomarkers. *Placenta* 29 691–698. 10.1016/j.placenta.2008.05.002 18573524

[B51] Herrera-Van Oostdam A. S. Salgado-Bustamante M. López J. A. Herrera-Van Oostdam D. A. López-Hernández Y. (2019). Placental exosomes viewed from an “omics” perspective: implications for gestational diabetes biomarkers identification. *Biomark. Med.* 13 675–684. 10.2217/bmm-2018-0468 31157549

[B52] Hiden U. Prutsch N. Gauster M. Weiss U. Frank H. G. Schmitz U. (2007). The first trimester human trophoblast cell line ACH-3P: a novel tool to study autocrine/paracrine regulatory loops of human trophoblast subpopulations – TNF-α stimulates MMP15 expression. *BMC Dev. Biol.* 7:137. 10.1186/1471-213X-7-137 18093301 PMC2263055

[B53] Hoang V. M. Foulk R. Clauser K. Burlingame A. Gibson B. W. Fisher S. J. (2001). Functional proteomics: examining the effects of hypoxia on the cytotrophoblast protein repertoire. *Biochemistry* 40 4077–4086. 10.1021/bi0023910 11300788

[B54] Horgan R. P. Broadhurst D. I. Dunn W. B. Brown M. Heazell A. E. P. Kell D. B. (2010). Changes in the metabolic footprint of placental explant-conditioned medium cultured in different oxygen tensions from placentas of small for gestational age and normal pregnancies. *Placenta* 31 893–901. 10.1016/j.placenta.2010.07.002 20708797

[B55] Horgan R. P. Kenny L. C. (2011). ‘Omic’ technologies: genomics, transcriptomics, proteomics and metabolomics. *Obstet. Gynaecol.* 13 189–195. 10.1576/toag.13.3.189.27672

[B56] Horii M. Li Y. Wakeland A. K. Pizzo D. P. Nelson K. K. Sabatini K. (2016). Human pluripotent stem cells as a model of trophoblast differentiation in both normal development and disease. *Proc. Natl. Acad. Sci. U.S.A.* 113 E3882–E3891. 10.1073/pnas.1604747113 27325764 PMC4941448

[B57] Horii M. Morey R. Bui T. Touma O. Nelson K. K. Cho H. Y. (2021). Modeling preeclampsia using human induced pluripotent stem cells. *Sci. Rep.* 11:5877. 10.1038/s41598-021-85230-5 33723311 PMC7961010

[B58] Hudecova I. Sahota D. Heung M. M. S. Jin Y. Lee W. S. Leung T. Y. (2014). Maternal plasma fetal DNA fractions in pregnancies with low and high risks for fetal chromosomal aneuploidies. *PLoS One* 9:88484. 10.1371/journal.pone.0088484 24586333 PMC3938419

[B59] Io S. Kabata M. Iemura Y. Semi K. Morone N. Minagawa A. (2021). Capturing human trophoblast development with naive pluripotent stem cells *in vitro*. *Cell Stem Cell* 28, 1023–1039. 10.1016/j.stem.2021.03.013 33831365

[B60] Ishihara N. Matsuo H. Murakoshi H. Laoag-Fernandez J. B. Samoto T. Maruo T. (2002). Increased apoptosis in the syncytiotrophoblast in human term placentas complicated by either preeclampsia or intrauterine growth retardation. *Am. J. Obstet. Gynecol.* 186 158–166. 10.1067/mob.2002.119176 11810103

[B61] Ishikawa A. Omata W. Ackerman W. E. Takeshita T. Vandré D. D. Robinson J. M. (2014). Cell fusion mediates dramatic alterations in the actin cytoskeleton, focal adhesions, and E-cadherin in trophoblastic cells. *Cytoskeleton* 71 241–256. 10.1002/cm.21165 24623684

[B62] Jaju Bhattad G. Jeyarajah M. J. McGill M. G. Dumeaux V. Okae H. Arima T. (2020). Histone deacetylase 1 and 2 drive differentiation and fusion of progenitor cells in human placental trophoblasts. *Cell Death Dis.* 11:311. 10.1038/s41419-020-2500-6 32366868 PMC7198514

[B63] Johnstone E. D. Sawicki G. Guilbert L. Winkler-Lowen B. Cadete V. J. J. Morrish D. W. (2011). Differential proteomic analysis of highly purified placental cytotrophoblasts in pre-eclampsia demonstrates a state of increased oxidative stress and reduced cytotrophoblast antioxidant defense. *Proteomics* 11 4077–4084. 10.1002/pmic.201000505 21800423

[B64] Karahalil B. (2016). Overview of systems biology and omics technologies. *Curr. Med. Chem.* 23 4221–4230. 27686657 10.2174/0929867323666160926150617

[B65] Karczewski K. J. Snyder M. P. (2018). Integrative omics for health and disease. *Nat. Rev. Genet.* 19 299–310. 10.1038/nrg.2018.4 29479082 PMC5990367

[B66] Karvas R. M. McInturf S. Zhou J. Ezashi T. Schust D. J. Roberts R. M. (2020). Use of a human embryonic stem cell model to discover GABRP, WFDC2, VTCN1 and ACTC1 as markers of early first trimester human trophoblast. *Mol. Hum. Reprod.* 26 425–440. 10.1093/molehr/gaaa029 32359161 PMC7320820

[B67] Kasak L. Rull K. Vaas P. Teesalu P. Laan M. (2015). Extensive load of somatic CNVs in the human placenta. *Sci. Rep.* 5 1–10. 10.1038/srep08342 25666259 PMC4914949

[B68] Kedia K. Nichols C. A. Thulin C. D. Graves S. W. (2015). Novel “omics” approach for study of low-abundance, low-molecular-weight components of a complex biological tissue: regional differences between chorionic and basal plates of the human placenta. *Anal. Bioanal. Chem* 407 8543–8556. 10.1007/s00216-015-9009-3 26350236

[B69] Kidima W. B. (2015). Syncytiotrophoblast functions and fetal growth restriction during placental malaria: updates and implication for future interventions. *Biomed. Res. Int.* 2015:451735. 10.1155/2015/451735 26587536 PMC4637467

[B70] Kliman H. J. Strauss J. F. Nestler J. E. Sermasi E. Strauss J. F. Sanger J. M. (1986). Purification, characterization, and in vitro differentiation of cytotrophoblasts from human term placentae. *Endocrinology* 118 1567–1582. 10.1210/endo-118-4-1567 3512258

[B71] Knöfler M. Haider S. Saleh L. Pollheimer J. Gamage T. K. J. B. James J. (2019). Human placenta and trophoblast development: key molecular mechanisms and model systems. *Cell. Mol. Life Sci.* 76 3479–3496. 10.1007/s00018-019-03104-6 31049600 PMC6697717

[B72] Knott J. G. Paul S. (2014). Transcriptional regulators of the trophoblast lineage in mammals with hemochorial placentation. *Reproduction* 148 R121–R136. 10.1530/REP-14-0072 25190503 PMC4231008

[B73] Kohler P. O. Bridson W. E. (1971). Isolation of hormone-producing clonal lines of human choriocarcinoma. *J. Clin. Endocrinol. Metab.* 32 683–687. 10.1210/jcem-32-5-683 5103722

[B74] Kolahi K. S. Valent A. M. Thornburg K. L. (2017). Cytotrophoblast, not syncytiotrophoblast, dominates glycolysis and oxidative phosphorylation in human term placenta. *Sci. Rep.* 7:42941. 10.1038/srep42941 28230167 PMC5322316

[B75] Konwar C. Del Gobbo G. Yuan V. Robinson W. P. (2019). Considerations when processing and interpreting genomics data of the placenta. *Placenta* 84 57–62. 10.1016/j.placenta.2019.01.006 30642669 PMC6612459

[B76] Krendl C. Shaposhnikov D. Rishko V. Ori C. Ziegenhain C. Sass S. (2017). GATA2/3-TFAP2A/C transcription factor network couples human pluripotent stem cell differentiation to trophectoderm with repression of pluripotency. *Proc. Natl. Acad. Sci. U.S.A.* 114 E9579–E9588. 10.1073/pnas.1708341114 29078328 PMC5692555

[B77] Kudo Y. Boyd C. A. R. Sargent I. L. Redman C. W. G. Lee J. M. Freeman T. C. (2004). An analysis using DNA microarray of the time course of gene expression during syncytialization of a human placental cell line (BeWo). *Placenta* 25 479–488. 15135230 10.1016/j.placenta.2003.12.001

[B78] Kumar P. Luo Y. Tudela C. Alexander J. M. Mendelson C. R. (2013). The c-Myc-Regulated MicroRNA-17∼92 (miR-17∼92) and miR-106a∼363 Clusters Target hCYP19A1 and hGCM1 To inhibit human trophoblast differentiation. *Mol. Cell. Biol.* 33 1782–1796. 10.1128/mcb.01228-12 23438603 PMC3624183

[B79] Kusama K. Bai R. Imakawa K. (2018). Regulation of human trophoblast cell syncytialization by transcription factors STAT5B and NR4A3. *J. Cell. Biochem.* 119 4918–4927. 10.1002/jcb.26721 29377304

[B80] Kwak Y. T. Muralimanoharan S. Gogate A. A. Mendelson C. R. (2019). Human trophoblast differentiation is associated with profound gene regulatory and epigenetic changes. *Endocrinology* 160 2189–2203. 10.1210/en.2019-00144 31294776 PMC6821221

[B81] Langbein M. Strick R. Strissel P. L. Vogt N. Parsch H. Beckmann M. W. (2008). Impaired cytotrophoblast cell-cell fusion is associated with reduced syncytin and increased apoptosis in patients with placental dysfunction. *Mol. Reprod. Dev.* 75 175–183. 10.1002/mrd.20729 17546632

[B82] Lavialle C. Cornelis G. Dupressoir A. Esnault C. Heidmann O. Vernochet C. (2013). Paleovirology of “syncytins”, retroviral env genes exapted for a role in placentation. *Philos. Trans. R. Soc. B Biol. Sci.* 368:20120507. 10.1098/rstb.2012.0507 23938756 PMC3758191

[B83] Lee C. Q. E. Gardner L. Turco M. Zhao N. Murray M. J. Coleman N. (2016). What Is Trophoblast? A Combination of Criteria Define Human First-Trimester Trophoblast. *Stem Cell Reports* 6 257–272. 10.1016/j.stemcr.2016.01.006 26862703 PMC4750161

[B84] Levine L. Habertheuer A. Ram C. Korutla L. Schwartz N. Hu R. W. (2020). Syncytiotrophoblast extracellular microvesicle profiles in maternal circulation for noninvasive diagnosis of preeclampsia. *Sci. Rep.* 10 1–11. 10.1038/s41598-020-62193-7 32286341 PMC7156695

[B85] Li H. Liu Y. Liu H. Sun X. (2020). Effect for human genomic variation during the bmp4-induced conversion from pluripotent stem cells to trophoblast. *Front. Genet.* 11:230. 10.3389/fgene.2020.00230 32318089 PMC7154154

[B86] Li Y. Moretto-Zita M. Soncin F. Wakeland A. Wolfe L. Leon-Garcia S. (2013). BMP4-directed trophoblast differentiation of human embryonic stem cells is mediated through a ΔNp63+ cytotrophoblast stem cell state. *Development.* 140 3965–3976. 10.1242/dev.092155 24004950 PMC3775414

[B87] Li Z. Kurosawa O. Iwata H. (2019). Establishment of human trophoblast stem cells from human induced pluripotent stem cell-derived cystic cells under micromesh culture. *Stem Cell Res. Ther.* 10:245. 10.1186/s13287-019-1339-1 31391109 PMC6686486

[B88] Liu Y. Ding D. Liu H. Sun X. (2017a). The accessible chromatin landscape during conversion of human embryonic stem cells to trophoblast by bone morphogenetic protein 4. *Biol. Reprod.* 96 1267–1278. 10.1093/biolre/iox028 28430877

[B89] Liu Y. Fan X. Wang R. Lu X. Dang Y. L. Wang H. (2018). Single-cell RNA-seq reveals the diversity of trophoblast subtypes and patterns of differentiation in the human placenta. *Cell Res.* 28 819–832. 10.1038/s41422-018-0066-y 30042384 PMC6082907

[B90] Liu Y. Yu S. Dhiman V. K. Brunetti T. Eckart H. White K. P. (2017b). Functional assessment of human enhancer activities using whole-genome STARR-sequencing. *Genome Biol.* 18:219. 10.1186/s13059-017-1345-5 29151363 PMC5694901

[B91] Lo Y. D. Leung T. N. Tein M. S. Sargent I. L. Zhang J. Lau T. K. (1999). Quantitative abnormalities of fetal DNA in maternal serum in preeclampsia. *Clin. Chem.* 45 184–188. 10.1093/clinchem/45.2.184 9931039

[B92] Lu X. Wang R. Zhu C. Wang H. Lin H. Y. Gu Y. (2017). Fine-tuned and cell-cycle-restricted expression of fusogenic protein syncytin-2 maintains functional placental syncytia. *Cell Rep.* 21 1150–1159. 10.1016/j.celrep.2017.10.019 29091755

[B93] Lv B. An Q. Zeng Q. Zhang X. Lu P. Wang Y. (2019). Single-cell RNA sequencing reveals regulatory mechanism for trophoblast cell-fate divergence in human peri-implantation conceptuses. *PLoS Biol.* 17:e3000187. 10.1371/journal.pbio.3000187 31596842 PMC6802852

[B94] Marchand M. Horcajadas J. A. Esteban F. J. McElroy S. L. Fisher S. J. Giudice L. C. (2011). Transcriptomic signature of trophoblast differentiation in a human embryonic stem cell model. *Biol. Reprod.* 84 1258–1271. 10.1095/biolreprod.110.086413 21368299

[B95] Mary S. Kulkarni M. J. Malakar D. Joshi S. R. Mehendale S. S. Giri A. P. (2017). Placental proteomics provides insights into pathophysiology of pre-eclampsia and predicts possible markers in plasma. *J. Proteome Res.* 16 1050–1060. 10.1021/acs.jproteome.6b00955 28030762

[B96] Mayhew T. M. (2014). Turnover of human villous trophoblast in normal pregnancy: what do we know and what do we need to know? *Placenta* 35 229–240. 10.1016/j.placenta.2014.01.011 24529666

[B97] Mayhew T. M. Barker B. L. (2001). Villous trophoblast: Morphometric perspectives on growth, differentiation, turnover and deposition of fibrin-type fibrinoid during gestation. *Placenta* 22 628–638. 10.1053/plac.2001.0700 11504531

[B98] Mayhew T. M. Leach L. McGee R. Wan Ismail W. Myklebust R. Lammiman M. J. (1999). Proliferation, differentiation and apoptosis in villous trophoblast at 13-41 weeks of gestation (including observations on annulate lamellae and nuclear pore complexes). *Placenta* 20 407–422. 10.1053/plac.1999.0399 10419806

[B99] McConkey C. A. Delorme-Axford E. Nickerson C. A. Kim K. S. Sadovsky Y. Boyle J. P. (2016). A three-dimensional culture system recapitulates placental syncytiotrophoblast development and microbial resistance. *Sci. Adv.* 2:e1501462. 10.1126/sciadv.1501462 26973875 PMC4783126

[B100] Meinhardt G. Haider S. Kunihs V. Saleh L. Pollheimer J. Fiala C. (2020). Pivotal role of the transcriptional co-activator YAP in trophoblast stemness of the developing human placenta. *Proc. Natl. Acad. Sci. U.S.A.* 117 13562–13570. 10.1073/pnas.2002630117 32482863 PMC7306800

[B101] Michelsen T. M. Henriksen T. Reinhold D. Powell T. L. Jansson T. (2019). The human placental proteome secreted into the maternal and fetal circulations in normal pregnancy based on 4-vessel sampling. *FASEB J.* 33 2944–2956. 10.1096/fj.201801193R 30335547

[B102] Miller R. K. Genbacev O. Turner M. A. Aplin J. D. Caniggia I. Huppertz B. (2005). Human placental explants in culture: approaches and assessments. *Placenta* 26 439–448. 10.1016/j.placenta.2004.10.002 15950058

[B103] Mischler A. Karakis V. Mahinthakumar J. Carberry C. K. Miguel A. S. Rager J. E. (2021). Two distinct trophectoderm lineage stem cells from human pluripotent stem cells. *J. Biol. Chem.* 296:100386. 10.1016/j.jbc.2021.100386 33556374 PMC7948510

[B104] Msheik H. El Hayek S. Bari M. F. Azar J. Abou-Kheir W. Kobeissy F. (2019). Transcriptomic profiling of trophoblast fusion using BeWo and JEG-3 cell lines. *Mol. Hum. Reprod.* 25 811–824. 10.1093/molehr/gaz061 31778538

[B105] Murphy V. E. Smith R. Giles W. B. Clifton V. L. (2006). Endocrine regulation of human fetal growth: the role of the mother, placenta, and fetus. *Endocr. Rev.* 27 141–169. 10.1210/er.2005-0011 16434511

[B106] Nalbantoglu S. Karadag A. (2019). “Introductory chapter: Insight into the OMICS technologies and molecular medicine,” in *Molecular Medicine*, ed. Nalbantoglu S. (London: IntechOpen), 10.5772/intechopen.86450

[B107] Nampoothiri L. P. Neelima P. S. Rao A. J. (2007). Proteomic profiling of forskolin-induced differentiated BeWo cells: an in-vitro model of cytotrophoblast differentiation. *Reprod. Biomed. Online* 14 477–487. 10.1016/S1472-6483(10)60896-6 17425831

[B108] Okae H. Toh H. Sato T. Hiura H. Takahashi S. Shirane K. (2018). Derivation of human trophoblast stem cells. *Cell Stem Cell* 22 50–63.e6. 10.1016/j.stem.2017.11.004 29249463

[B109] Orendi K. Gauster M. Moser G. Meiri H. Huppertz B. (2010). The choriocarcinoma cell line BeWo: syncytial fusion and expression of syncytium-specific proteins. *Reproduction* 140 759–766. 10.1530/REP-10-0221 20696850

[B110] Ouyang Y. Bayer A. Chu T. Tyurin V. A. Kagan V. E. Morelli A. E. (2016). Isolation of human trophoblastic extracellular vesicles and characterization of their cargo and antiviral activity. *Placenta* 47 86–95. 10.1016/j.placenta.2016.09.008 27780544 PMC5123854

[B111] Paradela A. Bravo S. B. Henríquez M. Riquelme G. Gavilanes F. González-Ros J. M. (2005). Proteomic analysis of apical microvillous membranes of syncytiotrophoblast cells reveals a high degree of similarity with lipid rafts. *J. Proteome Res.* 4 2435–2441. 10.1021/pr050308v 16335998

[B112] Pattillo R. A. Gey G. O. (1968). The Establishment of a cell line of human hormone-synthesizing trophoblastic cells in vitro. *Cancer Res.* 28 1231–1236. 4299001

[B113] Pattillo R. A. Ruckert A. C. F. Hussa R. O. Bernstein R. Delfs E. (1971). The JAr cell line – continuous human multi-hormone production and controls. *Vitr. Cell. Dev. Biol. Plant* 6 398–399.

[B114] Pavličev M. Wagner G. P. Chavan A. R. Owens K. Maziarz J. Dunn-Fletcher C. (2017). Single-cell transcriptomics of the human placenta: inferring the cell communication network of the maternal-fetal interface. *Genome Res.* 27 349–361. 10.1101/gr.207597.116 28174237 PMC5340963

[B115] Peñaherrera M. S. Jiang R. Avila L. Yuen R. K. C. Brown C. J. Robinson W. P. (2012). Patterns of placental development evaluated by X chromosome inactivation profiling provide a basis to evaluate the origin of epigenetic variation. *Hum. Reprod.* 27 1745–1753. 10.1093/humrep/des072 22431562 PMC3357192

[B116] Petroff M. G. Phillips T. A. Ka H. Pace J. L. Hunt J. S. (2006). Isolation and culture of term human trophoblast cells. *Methods Mol. Med* 121 203–217. 10.1385/1-59259-983-4:201 16251745

[B117] Poon L. C. Y. Musci T. Song K. Syngelaki A. Nicolaides K. H. (2013). Maternal plasma cell-free fetal and maternal DNA at 11-13 weeks’ gestation: relation to fetal and maternal characteristics and pregnancy outcomes. *Fetal Diagn. Ther.* 33 215–223. 10.1159/000346806 23466432

[B118] Qi W. H. Zheng M. Y. Li C. Xu L. Xu J. E. (2017). Screening of differential proteins of placenta tissues in patients with pre-eclampsia by iTRAQ proteomics techniques. *Minerva Med.* 108 389–395. 10.23736/S0026-4806.17.05080-7 28728340

[B119] Rafaeli-Yehudai T. Imterat M. Douvdevani A. Tirosh D. Benshalom-Tirosh N. Mastrolia S. A. (2018). Maternal total cell-free DNA in preeclampsia and fetal growth restriction: Evidence of differences in maternal response to abnormal implantation. *PLoS One* 13:e0200360. 10.1371/journal.pone.0200360 30001403 PMC6042756

[B120] Redman C. W. G. Staff A. C. (2015). Preeclampsia, biomarkers, syncytiotrophoblast stress, and placental capacity. *Am. J. Obstet. Gynecol.* 213 (4 Suppl.) S9.e1–S9.e4. 10.1016/j.ajog.2015.08.003 26428507

[B121] Renaud S. J. Chakraborty D. Mason C. W. Karim Rumi M. A. Vivian J. L. Soares M. J. (2015). OVO-like 1 regulates progenitor cell fate in human trophoblast development. *Proc. Natl. Acad. Sci. U.S.A.* 112 E6175–E6184. 10.1073/pnas.1507397112 26504231 PMC4653227

[B122] Roberts R. M. Loh K. M. Amita M. Bernardo A. S. Adachi K. Alexenko A. P. (2014). Differentiation of trophoblast cells from human embryonic stem cells: to be or not to be? *Reproduction* 147 D1–D12. 10.1530/REP-14-0080 24518070 PMC12802576

[B123] Roland C. S. Hu J. Ren C. E. Chen H. Li J. Varvoutis M. S. (2016). Morphological changes of placental syncytium and their implications for the pathogenesis of preeclampsia. *Cell. Mol. Life Sci.* 73 365–376. 10.1007/s00018-015-2069-x 26496726 PMC4846582

[B124] Rothbauer M. Patel N. Gondola H. Siwetz M. Huppertz B. Ertl P. (2017). A comparative study of five physiological key parameters between four different human trophoblast-derived cell lines. *Sci. Rep* 7 5892. 10.1038/s41598-017-06364-z 28724925 PMC5517571

[B125] Rouault C. Clément K. Guesnon M. Henegar C. Charles M. A. Heude B. (2016). Transcriptomic signatures of villous cytotrophoblast and syncytiotrophoblast in term human placenta. *Placenta* 44 83–90. 10.1016/j.placenta.2016.06.001 27452442

[B126] Saben J. Zhong Y. McKelvey S. Dajani N. K. Andres A. Badger T. M. (2014). A comprehensive analysis of the human placenta transcriptome. *Placenta* 35 125–131. 10.1016/j.placenta.2013.11.007 24333048 PMC3978120

[B127] Salomon C. Kobayashi M. Ashman K. Sobrevia L. Mitchell M. D. Rice G. E. (2013). Hypoxia-induced changes in the bioactivity of cytotrophoblast-derived exosomes. *PLoS One* 8:e79636. 10.1371/journal.pone.0079636 24244532 PMC3823597

[B128] Sarkar P. Mischler A. Randall S. M. Collier T. S. Dorman K. F. Boggess K. A. (2016). Identification of epigenetic factor proteins expressed in human embryonic stem cell-derived trophoblasts and in human placental trophoblasts. *J. Proteome Res.* 15 2433–2444. 10.1021/acs.jproteome.5b01118 27378238

[B129] Sarkar P. Randall S. M. Collier T. S. Nero A. Russell T. A. Muddiman D. C. (2015). Activin/nodal signaling switches the terminal fate of human embryonic stem cell-derived trophoblasts. *J. Biol. Chem.* 290 8834–8848. 10.1074/jbc.M114.620641 25670856 PMC4423675

[B130] Shankar K. Kang P. Zhong Y. Borengasser S. J. Wingfield C. Saben J. (2015). Transcriptomic and epigenomic landscapes during cell fusion in BeWo trophoblast cells. *Placenta* 36 1342–1351. 10.1016/j.placenta.2015.10.010 26515927

[B131] Sheridan M. A. Yang Y. Jain A. Lyons A. S. Yang P. Brahmasani S. R. (2019). Early onset preeclampsia in a model for human placental trophoblast. *Proc. Natl. Acad. Sci. U.S.A.* 116 4336–4345. 10.1073/pnas.1816150116 30787190 PMC6410818

[B132] Shi J. Feng H. Lee J. Chen W. N. (2013). Comparative proteomics profile of lipid-cumulating oleaginous yeast: an iTRAQ-coupled 2-D LC-MS/MS analysis. *PLoS One* 8:e85532. 10.1371/journal.pone.0085532 24386479 PMC3873444

[B133] Southcombe J. Tannetta D. Redman C. Sargent I. (2011). The immunomodulatory role of syncytiotrophoblast microvesicles. *PLoS One* 6:e20245. 10.1371/journal.pone.0020245 21633494 PMC3102084

[B134] Sudheer S. Bhushan R. Fauler B. Lehrach H. Adjaye J. (2012). FGF inhibition directs BMP4-mediated differentiation of human embryonic stem cells to syncytiotrophoblast. *Stem Cells Dev.* 21 2987–3000. 10.1089/scd.2012.0099 22724507 PMC3475151

[B135] Sun L. Yang N. De W. Xiao Y. (2007). Proteomic analysis of proteins differentially expressed in preeclamptic trophoblasts. *Gynecol. Obstet. Invest.* 64 17–23. 10.1159/000098399 17199091

[B136] Sun X. Qu T. He X. Yang X. Guo N. Mao Y. (2018). Screening of differentially expressed proteins from syncytiotrophoblast for severe early-onset preeclampsia in women with gestational diabetes mellitus using tandem mass tag quantitative proteomics. *BMC Pregnancy Childbirth* 18:437. 10.1186/s12884-018-2066-9 30404616 PMC6223002

[B137] Suryawanshi H. Morozov P. Straus A. Sahasrabudhe N. Max K. E. A. Garzia A. (2018). A single-cell survey of the human first-trimester placenta and decidua. *Sci. Adv.* 4:eaau4788. 10.1126/sciadv.aau4788 30402542 PMC6209386

[B138] Szilagyi A. Gelencser Z. Romero R. Xu Y. Kiraly P. Demeter A. (2020). Placenta-specific genes, their regulation during villous trophoblast differentiation and dysregulation in preterm preeclampsia. *Int. J. Mol. Sci.* 21:628. 10.3390/ijms21020628 31963593 PMC7013556

[B139] Taglauer E. S. Wilkins-haug L. Bianchi D. W. (2014). Review: cell-free fetal DNA in the maternal circulation as an indication of placental health and disease. *Placenta* 35 S64–S68. 10.1016/j.placenta.2013.11.014 24388429 PMC4886648

[B140] Tannetta D. Collett G. Vatish M. Redman C. Sargent I. (2017a). Syncytiotrophoblast extracellular vesicles – circulating biopsies reflecting placental health. *Placenta* 52 134–138. 10.1016/j.placenta.2016.11.008 27899180 PMC5423500

[B141] Tannetta D. Masliukaite I. Vatish M. Redman C. Sargent I. (2017b). Update of syncytiotrophoblast derived extracellular vesicles in normal pregnancy and preeclampsia. *J. Reprod. Immunol.* 119 98–106. 10.1016/j.jri.2016.08.008 27613663

[B142] Teasdale F. Jean-Jacques G. (1985). Morphometric evaluation of the microvillous surface enlargement factor in the human placenta from mid-gestation to term. *Placenta* 6 375–381. 10.1016/S0143-4004(85)80014-X 4070179

[B143] Than N. G. Romero R. Tarca A. L. Kekesi K. A. Xu Y. Xu Z. (2018). Integrated systems biology approach identifies novel maternal and placental pathways of preeclampsia. *Front. Immunol.* 9:1661. 10.3389/fimmu.2018.01661 30135684 PMC6092567

[B144] Tong M. Kleffmann T. Pradhan S. Johansson C. L. Desousa J. Stone P. R. (2016). Proteomic characterization of macro-, micro- and nano-extracellular vesicles derived from the same first trimester placenta: relevance for feto-maternal communication. *Hum. Reprod.* 31 687–699. 10.1093/humrep/dew004 26839151

[B145] Tsang J. C. H. Vong J. S. L. Ji L. Poon L. C. Y. Jiang P. Lui K. O. (2017). Integrative single-cell and cell-free plasma RNA transcriptomics elucidates placental cellular dynamics. *Proc. Natl. Acad. Sci. U.S.A.* 114 E7786–E7795. 10.1073/pnas.1710470114 28830992 PMC5604038

[B146] Tsuchida N. Kojima J. Fukuda A. Oda M. Kawasaki T. Ito H. (2020). Transcriptomic features of trophoblast lineage cells derived from human induced pluripotent stem cells treated with BMP 4. *Placenta* 89 20–32. 10.1016/j.placenta.2019.10.006 31675487

[B147] Turco M. Y. Gardner L. Kay R. G. Hamilton R. S. Prater M. Hollinshead M. S. (2018). Trophoblast organoids as a model for maternal–fetal interactions during human placentation. *Nature* 564 263–281. 10.1038/s41586-018-0753-3 30487605 PMC7220805

[B148] Van Patot M. C. T. Murray A. J. Beckey V. Cindrova-Davies T. Johns J. Zwerdlinger L. (2010). Human placental metabolic adaptation to chronic hypoxia, high altitude: hypoxic preconditioning. *Am. J. Physiol. Regul. Integr. Comp. Physiol.* 298 R166–R172. 10.1152/ajpregu.00383.2009 19864339 PMC2806207

[B149] Vandré D. D. Ackerman W. E. IV Tewari A. Kniss D. A. Robinson J. M. (2012). A placental sub-proteome: the apical plasma membrane of the syncytiotrophoblast. *Placenta* 33 207–213. 10.1016/j.placenta.2011.12.010 22222045 PMC3277652

[B150] Vento-Tormo R. Efremova M. Botting R. A. Turco M. Y. Vento-Tormo M. Meyer K. B. (2018). Single-cell reconstruction of the early maternal–fetal interface in humans. *Nature* 563 347–353. 10.1038/s41586-018-0698-6 30429548 PMC7612850

[B151] Walejko J. M. Chelliah A. Keller-Wood M. Gregg A. Edison A. S. (2018). Global metabolomics of the placenta reveals distinct metabolic profiles between maternal and fetal placental tissues following delivery in non-labored women. *Metabolites* 8:10. 10.3390/metabo8010010 29360753 PMC5876000

[B152] Wei Y. Zhou X. Huang W. Long P. Xiao L. Zhang T. (2017). Generation of trophoblast-like cells from the amnion in vitro: a novel cellular model for trophoblast development. *Placenta* 51 28–37. 10.1016/j.placenta.2017.01.121 28292466

[B153] West R. C. Ming H. Logsdon D. M. Sun J. Rajput S. K. Kile R. A. (2019). Dynamics of trophoblast differentiation in peri-implantation–stage human embryos. *Proc. Natl. Acad. Sci. U.S.A.* 116 22635–22644. 10.1073/pnas.1911362116 31636193 PMC6842583

[B154] Wice B. Menton D. Geuze H. Schwartz A. L. (1990). Modulators of cyclic AMP metabolism induce syncytiotrophoblast formation in vitro. *Exp. Cell Res.* 186 306–316. 10.1016/0014-4827(90)90310-7 2153559

[B155] Xu R. H. Chen X. Li D. S. Li R. Addicks G. C. Glennon C. (2002). BMP4 initiates human embryonic stem cell differentiation to trophoblast. *Nat. Biotechnol.* 20 1261–1264. 10.1038/nbt761 12426580

[B156] Yabe S. Alexenko A. P. Amita M. Yang Y. Schust D. J. Sadovsky Y. (2016). Comparison of syncytiotrophoblast generated from human embryonic stem cells and from term placentas. *Proc. Natl. Acad. Sci. U.S.A.* 113 E2598–E2607. 10.1073/pnas.1601630113 27051068 PMC4868474

[B157] Yang J. I. Kong T. W. Kim H. S. Kim H. Y. (2015). The proteomic analysis of human placenta with pre-eclampsia and normal pregnancy. *J. Korean Med. Sci.* 30 770–778. 10.3346/jkms.2015.30.6.770 26028931 PMC4444479

[B158] Yong H. E. J. Chan S. Y. (2020). Current approaches and developments in transcript profiling of the human placenta. *Hum. Reprod. Update* 26 799–840. 10.1093/humupd/dmaa028 33043357 PMC7600289

[B159] Yuan V. Hui D. Yin Y. Peñaherrera M. S. Beristain A. G. Robinson W. P. (2021). Cell-specific characterization of the placental methylome. *BMC Genomics* 22:6. 10.1186/s12864-020-07186-6 33407091 PMC7788826

[B160] Yuen R. K. C. Chen B. Blair J. D. Robinson W. P. Michael Nelson D. (2013). Hypoxia alters the epigenetic profile in cultured human placental trophoblasts. *Epigenetics* 8 192–202. 10.4161/epi.23400 23314690 PMC3592905

[B161] Zhang Q. Schulenborg T. Tan T. Lang B. Friauf E. Fecher-Trost C. (2010). Proteome analysis of a plasma membrane-enriched fraction at the placental feto-maternal barrier. *Proteomics Clin. Appl.* 4 538–549. 10.1002/prca.200900048 21137071

[B162] Zhang W. Lu S. Pu D. Zhang H. Yang L. Zeng P. (2019). Detection of fetal trisomy and single gene disease by massively parallel sequencing of extracellular vesicle DNA in maternal plasma: a proof-of-concept validation. *BMC Med. Genomics* 12:151. 10.1186/s12920-019-0590-8 31684971 PMC6829814

[B163] Zheng R. Li Y. Sun H. Lu X. Sun B. F. Wang R. (2017). Deep RNA sequencing analysis of syncytialization-related genes during BeWo cell fusion. *Reproduction* 153 35–48. 10.1530/REP-16-0343 27742864

[B164] Zhu C. Preissl S. Ren B. (2020). Single-cell multimodal omics: the power of many. *Nat. Methods* 17 11–14. 10.1038/s41592-019-0691-5 31907462

